# Context matters for the relationship between national identity and perceived democratic quality: National pride as a blind spot

**DOI:** 10.1111/bjso.70084

**Published:** 2026-04-29

**Authors:** Márton Hadarics

**Affiliations:** ^1^ Institute of Psychology ELTE Eötvös Loránd University Budapest Hungary

**Keywords:** democracy, national attachment, national identity, national pride, political support, social context

## Abstract

A growing body of evidence shows that national identity is positively related to attitudes toward societal and political systems. Yet much less is known about contextual factors that may modify this relationship. Distinguishing two facets of national identity—attachment and pride—and focusing on perceived democratic quality as a core system attitude, we test whether the links between these identity dimensions and system attitudes vary with the actual quality of democracy. Using data from 92 countries in the combined World Values Survey/European Values Study (*N* = 156,658), augmented with country‐level indicators, multilevel structural equation models show that the association between national pride and perceived democratic quality is stronger in less democratic countries, whereas the effect of attachment is context‐invariant. These findings suggest that national pride is associated with a positively biased perception of democratic quality that diverges from reality in illiberal or weak democracies, thereby complicating the predominantly positive framing of national pride in the social psychological literature on national identity.

## INTRODUCTION

A sense of shared group membership is widely regarded as a pillar of prosocial behavior and within‐group cooperation. This notion extends to whole societies, where recognition of a shared national identity is viewed as fundamental to the effective functioning of democratic systems. A shared national identity fosters mutual trust, cooperation and adherence to social norms (Huddy, 2023). Accordingly, national identity has been argued to enhance democratic quality by reinforcing the perceived legitimacy of democratic institutions and by fostering positive attitudes and trust toward them (Miller & Ali, 2014; Tamir, 1993). However, drawing primarily on social–psychological research on national identity (David & Bar‐Tal, 2009; Huddy, 2023) and system justification theory (Jost, 2019, 2020), we caution against an unreservedly positive view of national identity's role in democratic quality. We argue—and provide empirical evidence—that certain dimensions of national identity are associated with biased perceptions of democratic quality and processes, such as free and fair elections, particularly in contexts where the actual quality of these processes is questionable.

National identity is multifaceted, encompassing constructive or inclusive and destructive or chauvinistic forms of identification (Yogeeswaran & Verkuyten, 2022). In this study, we focus on national attachment and national pride and test their context‐dependent associations with perceived democratic and electoral quality. National attachment captures the subjective importance of national belonging, whereas national pride refers to pride in the nation's achievements (Huddy, 2023). Pride is a self‐evaluative emotion more directly linked to performance contexts and status signalling (Tracy & Robins, 2007; Williams & DeSteno, 2009). Owing to its evaluative nature, national pride may be more strongly related to evaluations of national democracy as a performance domain than is mere attachment, which lacks this direct evaluative component.

The interplay between national identity and perceived democratic quality may also be contingent on objective democratic performance. Recent findings in the system justification literature suggest that negative social contexts with severe social problems, like inequality or democratic deficit, can intensify both the motivational antecedents and the emotional consequences of perceived positive system performance (Hadarics & Kende, 2025; Napier et al., 2020). If national identity functions both as a relational motive and as an effective response to perceived high‐quality democratic functioning, its association with perceived democratic quality should be stronger when positive perceptions contradict a less democratic reality. Conversely, a more democratic context may impose reality constraints on positive perceptions, attenuating the effects of national identity. Whether such contextual catalysis operates similarly for attachment and pride remains an open question. If pride is more closely tied to evaluative perceptions (e.g., perceived democratic quality), its association may be more sensitive to these reality constraints.

### National Identity, attachment and pride

Belonging to positively evaluated groups and sustaining cooperative relations within them constitute fundamental social needs (Fiske, 2018). National identity—attachment to and identification with the national ingroup—addresses both. Yet national identification assumes multiple forms that differ in how explicitly they assert ingroup status. Consistent with Social Identity Theory (SIT; Tajfel & Turner, 1979), deriving self‐esteem from a positively valued group is a pervasive need and helps explain why even ostensibly benign identification, such as attachment, can yield mild ingroup bias (Huddy, 2023). Nevertheless, because attachment reflects the subjective importance of group belonging, it need not entail direct status comparisons with outgroups and can even predict positive outgroup attitudes when other identification forms are controlled (Cichocka, 2016). By contrast, chauvinistic forms—nationalism, national glorification (Roccas et al., 2006) and collective narcissism (Golec de Zavala, 2018)—are associated with stronger ingroup bias, often coupled with overt outgroup derogation, thereby explicitly assigning superior status to the ingroup.

National pride is often treated as a positive, constructive form of social identity that emphasizes affect toward the national ingroup (Huddy, 2023; Huddy & Del Ponte, 2019). Because pride rests on the nation's claimed achievements, status is likely more central to pride than to attachment, which does not directly reflect ingroup status. Research on pride as an affective experience characterizes it as a self‐reflective emotion arising from self‐evaluation of achievement; it promotes status seeking and signalling (Tracy et al., 2023). Pride both informs the individual and communicates to others a claim to approval and status within the group (Tracy & Robins, 2007; Williams & DeSteno, 2009). At the national level, pride lacks the overt superiority of chauvinistic or nationalist identification, yet it should relate more closely than attachment to status‐relevant evaluations of the nation. That attachment and pride are only partly overlapping facets of national identity is supported by evidence of divergent correlates: Huddy and Khatib (2007) reported that only attachment predicted voting, not pride, and Versteegen and Syropoulos (2025) found that priming shared national values increased attachment but not pride or other identity forms. These findings suggest that attachment is a relatively general identification dimension, whereas other forms, such as pride, possess distinctive features.

### National Identity, system‐attitudes and democracy

Because strong ingroup identification generally entails at least mild ingroup favouritism to satisfy needs for a positive social identity (Tajfel, 1981), it is unsurprising that national identification correlates with favourable attitudes toward the national social–political system, its democratic institutions and their functioning.

Most studies in this area do not differentiate among forms of national identification, so most evidence concerns attachment, often treated as national identity. Attachment correlates with indicators of system support, including general and economic system justification (Feygina et al., 2010; Moscato et al., 2021; Vargas‐Salfate et al., 2018), trust in democratic institutions (Caricati et al., 2021; Owuamalam et al., 2023) and satisfaction with institutional functioning (Owuamalam et al., 2023). Findings are more contradictory and context‐dependent for more chauvinistic identities. For instance, Vargas‐Salfate and Ayala (2020) reported a positive association between nationalism and system justification in Chile and Peru, whereas Austers et al. (2024) in Latvia found a negative association between nationalism and political trust; similarly, Gustavsson and Stendahl (2020) observed a negative association in the Netherlands but a positive one in the United States.

Evidence on the link between national pride and attitudes toward the political system is comparatively scarce. Gustavsson and Stendahl (2020) found that pride is positively related to political trust in both the Netherlands and the United States, and Mußotter and Rapp (2025) replicated this pattern across multiple Dutch panel datasets. Both studies also reported positive effects for attachment. Although neither explicitly compared the strengths of pride versus attachment, the point estimates for pride are larger in each case, supporting the view that pride is especially relevant when people evaluate their ingroup's performance in domains such as political or democratic functioning.

### Importance of the context

The system justification theory (SJT; Jost & Banaji, 1994; Jost, 2019, 2020) holds that, under certain conditions and motives, tendencies to judge social, political and economic systems as fair—and to perceive their functioning as appropriate—are strengthened. Although SIT and SJT disagree about the motivational bases that can lead even disadvantaged group members to view their status as legitimate, both acknowledge the importance of superordinate memberships, such as national identification, in shaping legitimacy perceptions (Jost, 2011; Jost et al., 2023; Rubin et al., 2023). The SIT approach of system attitudes explains the relationship with the need for a positively differentiated social identity, which identity establishes positive system attitudes (Rubin et al., 2023). On the other hand, the SJT approach describes strong national identity as an ideologically burdened embodiment of a more general system justification motive (Jost et al., 2023).

Compared with SIT, SJT research places greater emphasis on the role of social context in at least two respects. First, the epistemic and existential motives posited by SJT predict stronger associations with positive system perceptions when the system's actual functioning is more problematic. For example, belief in a just world and authoritarianism—motivated worldviews—relate more strongly to favorable attitudes toward the political system (e.g., satisfaction with and trust in democratic institutions) when democratic quality and performance are poorer (Hadarics & Kende, 2025; Hadarics & Krekó, 2025). Second, the unfavorable (vs. favorable) nature of context also shapes the emotional consequences of system‐justifying beliefs. SJT proposes that positive beliefs about the system serve a palliative function by alleviating distress and negative affect (Jost & Hunyady, 2003). Consistently, perceptions or beliefs that deny or justify significant social problems (e.g., income and racial inequalities, discrimination or deficits in democratic performance) correlate positively with indicators of positive affect such as happiness or life satisfaction, especially where these problems are more pronounced (Hadarics & Kende, 2025; Napier et al., 2010, 2020; Onraet et al., 2017; Rözer & Kraaykamp, 2013; Sengupta et al., 2017). Taken together, these findings imply that a negative social context—one that contradicts positive system attitudes—catalyzes both the effects of motivational antecedents and the affective consequences of system‐supportive perceptions. By contrast, in more favorable contexts, where such problems are absent or less severe, positive system attitudes may reflect accurate perception of reality; this “reality constraint” makes motivated and accurate perceptions harder to distinguish, helping to explain why motivational and affective correlates can be more weakly tied to system attitudes (Hadarics, 2024; Hadarics & Kende, 2025).

Context may matter for an additional reason in the link between national identification and democracy perceptions. Autocratic regimes often maintain the illusion of democratic functioning and reject accusations of violating core democratic principles (Bozóki, 2013; De Sa e Silva, 2022). Simultaneously, they tend to draw on nationalist and chauvinist ideological legitimation—while eschewing these labels in favor of terms such as patriotic, national‐conservative or national sovereignist (Bitschnau & Mußotter, 2024)—and frequently mobilize national pride in political communication and propaganda (Fabrykant & Magun, 2019; Gries, 2004). By equating regime with nation, these strategies allow propagandistic “democracy” narratives to blend with hubristic nationalism, furnishing communicative cues that encourage citizens to associate national identity with an illusion of democratic performance.

## THE STUDY

In this study, we test whether national pride exhibits a different relationship with perceived national democratic quality than national attachment, and how both relationships vary as a function of real democratic quality as a contextual moderator. Because pride is an affective experience closely tied to performance evaluation (e.g., national democratic performance), we hypothesized that pride would be more strongly associated with perceived democratic quality than attachment. Given evidence that negative contexts catalyze both the motivational bases and emotional consequences of system justification and pro‐system attitudes, we further expected the associations between both forms of national identification and perceived democratic quality to be stronger in less democratic countries. Finally, although attachment can also produce mild ingroup bias, we anticipated that contextual moderation by real democratic performance would be stronger for pride because of its closer fit to performance‐relevant judgements.

To test these hypotheses, we analyzed data from the World Values Survey (WVS) and European Values Study (EVS), supplemented with indicators from the Varieties of Democracy (V‐Dem) database. Perceived democratic quality was examined in two forms: (a) a general assessment of democratic quality and (b) perceptions of the quality, fairness and integrity of national elections. Although democratic procedures encompass a broad set of elements, free and fair elections are widely regarded—across diverse cultural contexts—as a core criterion for classifying a regime as democratic (Chu et al., 2024; Robbins & Tessler, 2012). Correspondingly, perceptions of electoral fairness are among the most influential predictors of political support and institutional trust, as properly functioning elections confer legitimacy (Mauk, 2022; Norris, 2019).

### Databases

Both the WVS and EVS are large‐scale international survey programs designed to map individuals' beliefs, values and attitudes on a wide range of social, cultural and political issues. Due to the substantial overlap in their item pools, combining responses from both programs yields a uniquely comprehensive dataset that captures public opinion across an exceptionally diverse cultural spectrum. The most recent combined WVS–EVS dataset—merging the 7th wave of the WVS and the 5th wave of the EVS (2017–2023)—includes responses from probabilistic representative samples in 92 countries and/or autonomous territories (*N* = 156,658), making it the most extensive international survey database available (EVS/WVS, 2022). To assess actual democratic quality, we used a variety of indices from the V‐Dem database, which ranks countries based on multiple benchmarks of democratic functioning.

### Variables

#### National Identity (WVS/EVS)

The two forms of national identification were assessed by two items, one tapping into national attachment (“How close do you feel to your [COUNTRY]?”; 1–4 scale) and the other into national pride (“How proud are you to be a [COUNTRY] citizen?”; 1–4 scale).

#### Perceived democratic quality (WVS/EVS)

For measuring perceived general democratic quality, we applied a single‐item indicator (“How democratically is this country being governed today?”; 1–10 scale), and for measuring perceived electoral quality, three items were selected (“Votes are counted fairly.”; “Journalists provide fair coverage of elections.”; “Election officials are fair.”; 1–4 scales).

#### Democratic quality

The actual quality of democracy was operationalized by the democracy indices of the V‐Dem database (V‐Dem Institute, 2025). These indices benchmark countries along a 0–1 scale for five conceptually different types of democratic functioning (liberal, electoral, egalitarian, participatory, deliberative).^1^ Based on standardized expert ratings and objective legislative and statistical data, these indices rely on more than 500 indicators to quantify democratic quality along the five dimensions. This multidimensional scoring system enables accounting for the complexity in the interpretations of both political theorizing and lay people about the concept of democracy (V‐Dem Institute, 2025). We used the V‐Dem indices from the years of the WVS data collections for each country.

#### Control variables

From the WVS/EVS database, respondents' gender (1 = male; 2 = female), age, education level (1 = lower; 2 = middle; 3 = upper), left–right ideology preference (1 = left; 10 = right), religiousness (1 = atheist; 2 = not religious; 3 = religious) and confidence in the incumbent government (1 = none at all confident; 4 = a great deal confident) were applied as control variables in the subsequent statistical analysis. As previous results show, social status, ideology and religiousness predict system attitudes (Jost, 2019, 2020; Mayne & Hakhverdian, 2017). Confidence in the government was introduced to control for partisan bias when predicting perceptions of democratic quality. We also applied societal development as a control variable in the form of the Human Development Index, which indicates on a 0–1 scale how effective the political–institutional system is at providing a long and healthy life, knowledge and a high standard of living for its citizens (United Nations, 2025). Descriptive statistics for the variables in the complete dataset are reported in Table 1, and descriptives for the participating countries are reported in Data S1.

**TABLE 1:** Descriptive statistics and estimated within‐ and between‐level variances.

	Mean	Variance	Estimated within variance	SE	*p*	Estimated between variance	SE	*p*	ICC
Model 1									
Perceived general democratic quality	6.186	6.825	5.503	0.214	<.001	1.271	0.152	<.001	0.187
National pride	3.434	0.548	0.472	0.018	<.001	0.078	0.009	<.001	0.144
National attachment	3.282	0.599	0.530	0.018	<.001	0.074	0.012	<.001	0.123
Gender	1.535	0.249	0.247	0.001	<.001	0.002	0.000	<.001	0.008
Age	45.118	294.140	258.578	6.273	<.001	37.560	4.229	<.001	0.127
Education	2.044	0.615	0.514	0.013	<.001	0.098	0.011	<.001	0.161
Religion	2.539	0.424	0.316	0.018	<.001	0.100	0.018	<.001	0.240
Left–right position	5.614	5.762	5.468	0.221	<.001	0.298	0.065	<.001	0.055
Confidence in government	2.329	0.919	0.698	0.021	<.001	0.211	0.030	<.001	0.231
Human Development Index	0.810	0.012	—	—	—	—	—	—	—
V‐Dem Electoral Democracy	0.592	0.065	—	—	—	—	—	—	—
V‐Dem Liberal Democracy	0.484	0.075	—	—	—	—	—	—	—
V‐Dem Participatory Democracy	0.401	0.044	—	—	—	—	—	—	—
V‐Dem Deliberative Democracy	0.474	0.066	—	—	—	—	—	—	—
V‐Dem Egalitarian Democracy	0.469	0.062	—	—	—	—	—	—	—
Model 2–34 countries									
‘Votes counted fairly’	3.040	1.047	0.650	0.047	<.001	0.385	0.056	<.001	0.369
‘Journalists are fair’	2.644	0.819	0.766	0.034	<.001	0.052	0.013	<.001	0.063
‘Election officials are fair’	2.893	0.975	0.702	0.040	<.001	0.261	0.039	<.001	0.267
National pride	3.392	0.575	0.504	0.029	<.001	0.073	0.016	<.001	0.131
National attachment	3.312	0.556	0.501	0.026	<.001	0.067	0.019	<.001	0.118
Gender	1.544	0.248	0.246	0.001	<.001	0.002	0.001	<.001	0.008
Age	47.222	307.658	276.745	8.112	<.001	36.504	7.822	.002	0.116
Education	2.120	0.579	0.515	0.027	<.001	0.077	0.019	<.001	0.131
Religion	5.435	5.430	5.135	0.363	<.001	0.340	0.152	.050	0.061
Left–right position	2.494	0.466	0.366	0.026	<.001	0.102	0.032	.002	0.217
Confidence in government	2.220	0.828	0.653	0.025	<.001	0.203	0.050	<.001	0.236
V‐Dem Electoral Democracy	0.657	0.057	—	—	—	—	—	—	—
Human Development Index	0.823	0.015	—	—	—	—	—	—	—

Abbreviations: ICC, intraclass correlation coefficient; SE, standard error.

### Analysis & Results

To test our hypotheses, we employed multilevel structural equation modeling (MSEM) with cross‐level interactions. A multilevel approach to data analysis is required when nested data (e.g., respondents within countries) violate the independence assumption by sharing context‐level influences. In such cases, multilevel models are needed to partition variance across levels, produce correct standard errors and estimate both within‐cluster and between‐cluster (including cross‐level) effects, which would mean within‐country and between‐country variances for the WVS–EVS variables in our case. In a multilevel model, the ‘between‐level’ indicates how groups (e.g., countries) differ in their average outcomes and possibly slopes, while the ‘within‐level’ is based on how individual observations vary around their own group's expected outcome after accounting for those group differences (Finch & Bolin, 2017). In Table 1, we report the results of a variance decomposition analysis along with intraclass correlation coefficients (ICC) for the WVS/EVS variables. Both the ICC values and the significant between‐level variances showed that these items varied systematically not only across individual respondents but also across countries, necessitating a multilevel approach to data analysis.

All analyses were conducted using full‐information Bayesian estimation, which provides more reliable estimates than traditional frequentist approaches, particularly with the sample sizes and data structures typical of multinational datasets (Finch & Bolin, 2017; Hox et al., 2012). Besides, the multi‐iteration approach of Bayesian estimation enabled the direct test of equality of the regression coefficients for attachment and pride, as it provides a distribution for the estimated difference for each iteration (Asparouhov & Muthén, 2010). Each model was estimated using two Markov chain Monte Carlo (MCMC) chains and 30,000 iterations, with the first half designated as the burn‐in phase. The burn‐in phase discards early draws, while chains move from arbitrary initial values toward the high‐probability posterior estimates. The number of iterations proved appropriate, since in each case our models converged successfully, as indicated by proportional scale reduction (PSR) factor values falling below 1.05 during the burn‐in phases and remaining below it throughout the estimation process (Vats & Knudson, 2021). We used *N* (0; ∞) priors for the slope estimates, which is a flexible choice if there are no well‐grounded prior assumptions about the exact strength of the estimates (Asparouhov & Muthén, 2010). Each analysis was performed with the MPlus 8.6 software.

We built two MSEM models, one with perceived general democratic quality and another with perceived electoral quality as the dependent variable. In the first model, the within‐level variance of perceived general democratic quality (variance between respondents regardless of their nationality) was predicted by national attachment, pride and the individual‐level control variables, while the between‐level variance (variances between countries) of the dependent variable was predicted by the country‐level variables of HDI and a latent variable based on the shared covariances of the five V‐Dem democracy indices, which we called democratic quality. A confirmatory factor analysis (CFA) model showed an excellent fit for this latent variable (CFI = 0.966; RMSEA = 0.066; SRMR = 0.004), which supports the appropriate psychometric qualities of it.

In the second model, the dependent variable was a latent variable based on the shared within‐level covariances of the three indicator items of perceived electoral quality. Since respondents were from several countries, we tested the measurement invariance of this latent variable with an alignment optimization test, which is an appropriate invariance testing approach for nested data structures with a high number of clusters (Asparouhov & Muthén, 2023).^2^ The alignment test flagged 34 countries, in which case the factor loadings of the three items were invariant. Data only from these countries were included in the second MSEM model, as non‐invariance signals that the interpretation and/or meaning of the latent construct varies and is different for respondents from the excluded countries. More detailed results of the alignment test can be found in Data S1, along with the list of the 34 countries included in the second MSEM model. A subsequent multigroup CFA model with the 34 countries showed a perfect fit for the latent variable (CFI = 1.00; RMSEA = 0.00; SRMR = 0.00).

For both final MSEM models, the within‐level predictors were group‐mean‐centered, and the between‐level predictors and moderators were grand‐mean‐centered. In both models, the effects of the two forms of national identity were defined as random slopes, which could vary between countries. Simultaneously, on the between‐level of the models, the between‐country variances of these random slopes were correlated with the country‐level moderators—the HDI and the democratic quality indicators—to build cross‐level interactions, which could indicate whether contextual democratic quality moderates the statistical effects of the national identity variables on the within‐level perceived democratic quality variables. We used only the Electoral Democracy Index (EDI) from the V‐Dem indices as a between‐level moderator for the model predicting within‐level perceived electoral quality because only this showed a conceptual fit with the dependent variable, as the latter indicates the subjective perception of free and fair elections, while the EDI shows its actual contextual functioning. Furthermore, it is also important to highlight that in this second model, the HDI and the EDI were applied only as moderators, but not as direct predictors at the between‐level. The reason for this is that the dependent variable, the latent variable of perceived electoral quality, was constructed on the within‐level of the model. In a multilevel setting, any variable can correlate with other variables on the same level; consequently, the between‐level indices could not predict perceived electoral quality on the within‐level but, as moderators, could predict the between‐level variances of the random slopes for the two national identity variables.

**FIGURE 1:**
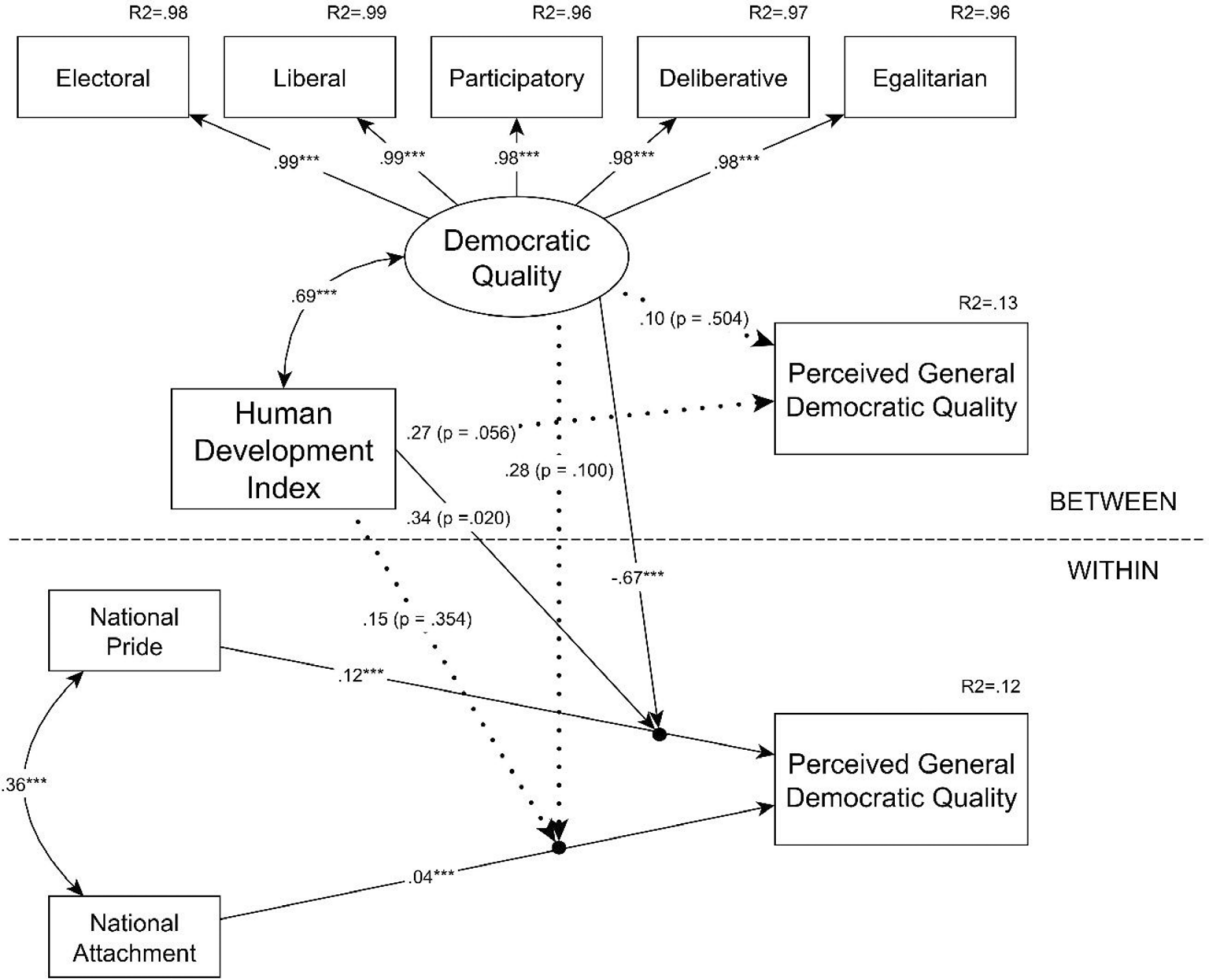
Multilevel SEM model predicting perceived general democratic quality. Control variables are not displayed. ***p < .001.

Details of the model predicting perceived general democratic quality are reported in Table 2, and the most important results can be seen in Figure 1. These show that both forms of national identification had a positive statistical effect on the within‐level perceived general democratic quality (attachment: *b* = 0.13; *p* < .001; pride: *b* = 0.39; *p* < .001), and the effect of pride was significantly stronger (Δ*b* = 0.26; *SD* = 0.04; 95% CI: [0.18; 0.34]; *p* < .001). Furthermore, from these two effects, only the effect of national pride was moderated by the between‐level moderators (democratic quality: *b* = −0.17; *p* < .001; HDI: *b* = 0.77; *p* = .020). Subsequent simple slope analyses showed that the relationship between pride and perceived democratic quality was weaker at a high level (+1 *SD*) of democratic quality and turned into non‐significant at a very high level (+2 *SD*) of the moderator. In comparison, the relationship got stronger at lower levels (−1 *SD* and −2 *SD*) of democratic quality. Interestingly, in the case of the HDI, the pattern was the opposite, as the effect of pride was stronger at higher levels (+1 *SD* and +2 *SD*) of the moderator and weaker at lower levels (−1 *SD* and −2 *SD*) of it. Details of the simple slope analyses are reported in Table  4.

**TABLE 2:** Multilevel SEM model predicting perceived general democratic quality.

	Standardized estimate	Unstandardized estimate	*SD*	95% CI LB	95% CI UB	*p*
Within‐level						
Regression coefficients						
National attachment	0.041	0.129	0.019	0.092	0.167	<.001
National pride	0.115	0.390	0.031	0.328	0.452	<.001
Gender	0.004	0.019	0.012	−0.005	0.043	.124
Age	−0.006	−0.001	0.000	−0.002	0.000	.028
Education	0.021	0.067	0.009	0.050	0.084	<.001
Left–right position	0.083	0.082	0.003	0.076	0.088	<.001
Religion	−0.001	−0.005	0.011	−0.026	0.017	.678
Confidence in government	0.286	0.797	0.007	0.782	0.811	<.001
Between‐level						
Factor weights (general democratic quality)						
V‐Dem Electoral Democracy	0.988	0.266	0.019	0.235	0.307	<.001
V‐Dem Liberal Democracy	0.996	0.288	0.020	0.255	0.331	<.001
V‐Dem Participatory Democracy	0.979	0.216	0.015	0.190	0.250	<.001
V‐Dem Deliberative Democracy	0.983	0.267	0.019	0.235	0.308	<.001
V‐Dem Egalitarian Democracy	0.978	0.257	0.018	0.226	0.297	<.001
Regression coefficients						
General Democratic Quality	0.095	0.111	0.171	−0.225	0.453	.504
Human Development Index	0.270	2.752	1.435	−0.087	5.570	.056
Cross‐level interactions						
National attachment × General Democratic Quality	0.276	0.045	0.028	−0.009	0.101	.100
National attachment × Human Development Index	0.152	0.215	0.230	−0.237	0.668	0.354
National pride × General Democratic Quality	−0.666	−0.171	0.040	−0.252	−0.095	<.001
National pride × Human Development Index	0.343	0.768	0.324	0.129	1.401	.020
*R* ^2^ values						
Perceived General Democratic Quality (within‐level)	0.115	—	0.002	0.112	0.118	<.001
Perceived General Democratic Quality (between‐level)	0.129	—	0.063	0.029	0.274	<.001
National attachment (random slope)	0.171	—	0.085	0.035	0.359	<.001
National pride (random slope)	0.257	—	0.091	0.095	0.449	<.001

Abbreviations: 95% CI LB, lower bounds of the 95% Bayesian credibility interval; 95% CI UB, upper bounds of the 95% Bayesian credibility interval; *SD*, posterior standard deviation.

**FIGURE 2:**
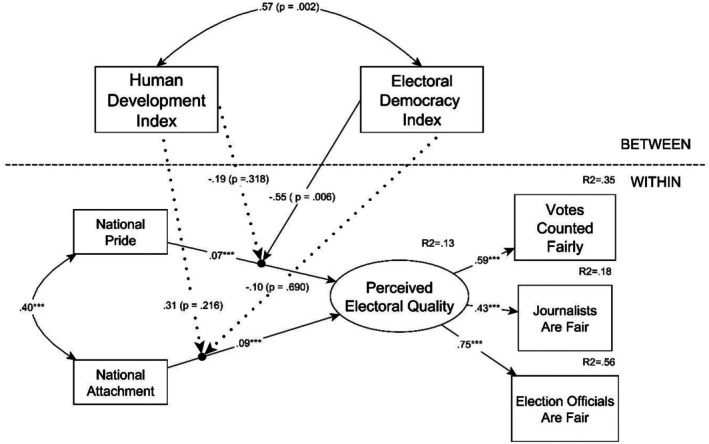
Multilevel SEM model predicting perceived electoral quality. Control variables are not displayed. ***p < .001.

Details of the model predicting perceived electoral quality are reported in Table 3, and the most important results can be seen in Figure 2. These show that both forms of national identification had a positive statistical effect on the within‐level perceived electoral quality (attachment: *b* = 0.14; *p* < .001; pride: *b* = 0.11; *p* < .001), and there was no significant difference between these effects (Δ*b* = 0.03; *SD* = 0.03; 95% CI: [−0.03; 0.09]; *p* = .294). Furthermore, from these two effects, only the effect of national pride was moderated and only by the EDI (*b* = −0.30; *p* = .006). Subsequent simple slope analyses showed that the relationship between pride and perceived electoral quality was non‐significant at higher levels (+1 *SD* and +2 *SD*) of the EDI. Nonetheless, compared to the main effect, the relationship got stronger at lower levels (−1 *SD* and −2 *SD*) of the moderator. Details of the simple slope analyses are reported in Table 4.

**TABLE 3:** Multilevel SEM model predicting perceived electoral democratic quality.

	Standardized estimate	Unstandardized estimate	*SD*	95% CI LB	95% CI UB	*p*
Within‐level						
Factor weights (Perceived electoral quality)						
‘Votes counted fairly’	0.590	0.444	0.004	0.436	0.452	<.001
‘Journalists are fair’	0.425	0.348	0.004	0.339	0.356	<.001
‘Election officials are fair’	0.745	0.582	0.005	0.572	0.592	<.001
Regression weights						
National attachment	0.091	0.138	0.020	0.097	0.178	<.001
National pride	0.068	0.106	0.021	0.064	0.148	<.001
Gender	−0.029	−0.064	0.011	−0.086	−0.042	<.001
Age	0.090	0.006	0.000	0.005	0.006	<.001
Education	0.082	0.122	0.008	0.106	0.137	<.001
Left–right position	−0.016	−0.008	0.003	−0.013	−0.002	.006
Religion	−0.012	−0.021	0.010	−0.040	−0.002	.028
Confidence in government	0.287	0.380	0.007	0.366	0.395	<.001
Between‐level						
Cross‐level interactions						
National attachment × V‐Dem Electoral Democracy	−0.095	−0.040	0.106	−0.251	0.169	.690
National attachment × Human Development Index	0.305	0.242	0.198	−0.148	0.633	.216
National pride × V‐Dem Electoral Democracy	−0.553	−0.304	0.108	−0.519	−0.093	.006
National pride × Human Development Index	−0.186	−0.196	0.200	−0.586	0.206	.318
*R* ^2^ values						
Perceived Electoral Quality (Within‐level)	0.127	—	0.004	0.121	0.135	<.001
National attachment (random slope)	0.116	—	0.108	0.005	0.403	<.001
National pride (random slope)	0.479	—	0.149	0.164	0.737	<.001

Abbreviations: 95% CI LB, lower bounds of the 95% Bayesian credibility interval; 95% CI UB, upper bounds of the 95% Bayesian credibility interval; *SD*, posterior standard deviation.

**TABLE 4:** Simple slope analyses of the relationship between national pride and perceived democratic quality.

	Moderator	Level	Unstandardized estimate	*SD*	95% CI LB	95% CI UB	*p*
Model 1	General democratic quality	+2 *SD*	0.049	0.086	−0.126	0.212	.570
		+1 *SD*	0.220	0.051	0.115	0.315	<.001
		Mean	0.390	0.031	0.328	0.452	<.001
		−1 *SD*	0.560	0.050	0.467	0.666	<.001
		−2 *SD*	0.731	0.085	0.571	0.907	<.001
	Human Development Index	+2 *SD*	0.559	0.077	0.409	0.712	<.001
		+1 *SD*	0.474	0.047	0.385	0.568	<.001
		Mean	0.390	0.031	0.328	0.452	<.001
		−1 *SD*	0.307	0.048	0.210	0.399	<.001
		−2 *SD*	0.222	0.079	0.065	0.375	.006
Model 2	V‐Dem Electoral Democracy	+2 *SD*	−0.039	0.055	−0.146	0.069	.468
		+1 *SD*	0.034	0.033	−0.031	0.098	.298
		Mean	0.106	0.021	0.064	0.148	<.001
		−1 *SD*	0.179	0.034	0.112	0.246	<.001
		−2 *SD*	0.251	0.056	0.142	0.364	<.001

Abbreviations: 95% CI LB, lower bounds of the 95% Bayesian credibility interval; 95% CI UB, upper bounds of the 95% Bayesian credibility interval; *SD*, posterior standard deviation.

## DISCUSSION

Our results showed that both national attachment and pride had a positive relationship with both indicators of perceived democratic quality, general democratic quality on the one hand and electoral democratic quality on the other. However, pride had a stronger statistical effect only in the first case, but the two effects were equal in the latter case. Furthermore, contextual democratic quality was not related to the effect of attachment in either case, but affected how strongly pride was related to perceived democratic quality in both cases, in a way that the relationship is stronger in less democratic countries.

The finding that both forms of national identification are related to positive perceptions of national democracy is in line with both the SIT (Tajfel & Turner, 1979) and the SIT‐based interpretations of national identity (e.g., Caricati et al., 2021; Gustavsson & Stendahl, 2020; Owuamalam et al., 2023), which emphasize that the need for a positive national identity is related to at least a mild form of ingroup bias. Since people want to belong to valuable groups, it is not surprising that stronger identification correlates with more positive ingroup perceptions. Nonetheless, since pride, as an affective factor, is more relevant in performance and achievement contexts (Tracy et al., 2023), we assumed that it is more closely related to perceived democratic quality than attachment. Although without explicitly testing, previous studies also pointed in this direction (Gustavsson & Stendahl, 2020; Mußotter & Rapp, 2025). However, our results are inconclusive in this regard, as we found supporting evidence for perceived general democratic quality, but not for a more specific evaluative dimension, electoral democratic quality. However, it is worth noting that the two cases differ in both the scope of the analyzed datasets and the generality (vs. specificity) of the two evaluative dimensions (general vs. electoral democratic quality). Unfortunately, we had to exclude the majority of the participating countries from our second model due to the metric non‐invariance of the electoral democratic quality latent variable; consequently, the results of this model are based on a much more restricted geographical scope than the first model. Secondly, it is also worth taking into account that this latent variable was based on relatively specific opinions about the electoral quality, while the democratic quality item in the first model was more general. It is possible that this general item was interpreted in a more diffuse (vs. specific) way by the respondents, which enables emotionally charged and motivated responses to a greater extent than items asking about more specific opinions (Easton, 1975; Norris, 2017). Under this interpretation, it is understandable that pride—an affective, motivational factor in performance judgments—showed a stronger effect for the more diffuse item. Nevertheless, further investigation is required to test the differential effects of attachment and pride.

A consistent result across the two models was that the actual democratic quality as a contextual characteristic affected only the relationship between perceived democratic quality and pride, but not attachment. It seems that attachment is associated with more positive perceptions of democratic functioning, regardless of the level of actual democratic functioning. In the case of pride, however, contextual democratic deficit has the potential to catalyze its association with positive perceptions of democratic quality. This is in line with the results of other former studies from the SJT literature, which show that both the motivational antecedents and the emotional consequences of positive system attitudes and perceptions are stronger in negative, more problematic contexts that contradict these attitudes and perceptions, but are weaker in positive contexts where a reality constraint makes positive perceptions more independent from these antecedents and consequences (e.g., Hadarics & Krekó, 2025; Napier et al., 2020).

However, whether national pride is a motivational base or an emotional consequence of positive perceptions of democratic quality, or both, is still unclear, given the cross‐sectional nature of our study. Despite their disagreements about the scope of potential motivational foundations of positive system attitudes, both SIT and the SJT emphasize that the need for a positive social identity, or group‐justification, can be an important motivational base for positive system attitudes, especially when higher‐level social identities, like national identity, are more available than others (Jost, 2011; Rubin et al., 2023). This interpretation would describe pride more as a relational motivational factor driving biased perceptions of democratic quality and would imply that national pride makes people perceive democratic functioning positively, especially in countries with more severe democratic deficits. On the other hand, pride, as an individual‐level emotion, is usually described as an affective reaction to personal accomplishments (Tracy et al., 2023), which would position national pride as an emotional consequence of positive perceptions of democratic quality, especially in contexts where these perceptions are not accurate. It is also important to emphasize that these two possibilities are not mutually exclusive, and national pride might serve in both functions simultaneously. Further experimental and longitudinal research is needed to disentangle the possible two‐way causal directions between national pride and system attitudes. However, the fact that national pride is related to unwarranted and biased positive perceptions of democratic functioning—especially in ill‐democracies—goes against the usually positive role that has been assigned to national pride so far in modern democratic functioning (Gustavsson & Stendahl, 2020; Huddy, 2023).

Although we made no prior assumption on this point, our results indicate that, controlling for actual democratic quality, the association between national pride and perceived general democratic quality is stronger in more developed countries. While this may appear counterintuitive, it accords with the political‐support literature on performance legitimacy (e.g., Levi, 2018; Nathan, 2020), whereby citizens ground support in the state's economic, welfare and service performance rather than (or in addition to) procedural criteria. Given that understandings of “democracy” are diverse and sometimes incorporate materialist performance dimensions (Canache, 2012; Dalton et al., 2007), it is unsurprising that national pride can rest on such achievements. By contrast, no such moderation emerged for perceived electoral quality, where the indicator items afforded substantially less scope for subjective interpretation by focusing on the procedural criterion of free and fair elections.

A limitation of our study is that we examined only two forms of national identification—attachment and pride—because the WVS/EVS lack items tapping more chauvinistic variants such as nationalism, glorification or collective narcissism. As noted above, findings on the links between these forms and positive system attitudes are contradictory (e.g., Austers et al., 2024; Gustavsson & Stendahl, 2020; Vargas‐Salfate & Ayala, 2020), underscoring the need to test contextual moderators systematically. It is also noteworthy that autocratic and illiberal regimes often seek legitimacy and support through nationalist and/or chauvinistic ideologies while disavowing those labels and allegations of democratic abuse (Krekó & Enyedi, 2018; von Soest & Grauvogel, 2017). Because such regimes pair nationalism with denial of democratic backsliding in their propaganda, the association between nationalistic identities and perceived democratic quality may be stronger in these contexts.

Jointly examining national pride and nationalism within a context‐sensitive framework would be especially informative, as these dimensions may map onto distinct forms of pride. Tracy et al. (2023) suggest that, at the collective level, national pride aligns with authentic pride—a genuine sense of accomplishment and self‐worth—implying pride in real or assumed national achievements. By contrast, national chauvinism appears closer to hubristic pride, a more self‐aggrandizing, entitlement‐focused stance. In the case of nationalism, this implies group‐based dominance and generalized superiority, traits found to correlate with chauvinistic national identities (Cichocka, 2016; Huddy, 2023; Yogeeswaran & Verkuyten, 2022). If these two forms of pride operate at the collective level, future research should more finely differentiate their psychological functioning and contextual dependencies.

## CONCLUSION

Strong national identity and attachment to the national group are often posited as foundations of ingroup solidarity, cooperation and trust—core requisites of a well‐functioning democracy (Miller & Ali, 2014). Alongside attachment, national pride is frequently treated as a positive, constructive dimension of identification, emphasizing the favorable affect attached to the national ingroup (Huddy, 2023). Our results, however, indicate a darker side: with respect to perceptions of democratic functioning and the rule of law, pride can be associated with unwarrantedly positive system evaluations. In countries with more severe democratic problems, national pride correlates with groundless, positively biased assessments of the system, raising the possibility that pride functions as a blind spot for democratic deficit.

## AUTHOR CONTRIBUTIONS

**Márton Hadarics:** Conceptualization; methodology; investigation; data curation; formal analysis; visualization; project administration; resources; writing – original draft.

## FUNDING INFORMATION

This project was supported by the János Bolyai Research Scholarship of the Hungarian Academy of Sciences (grant number: BO/00662/25/2). The research was conducted within the framework of the project MORES—Moral Emotions in Politics: How They Unite, How They Divide, which has received funding from the European Union under Grant Agreement No. 101132601.

## CONFLICT OF INTEREST STATEMENT

The author declare no conflicts of interest.

## ETHICS STATEMENT

This study required no ethics approval.

## Supporting information


Data S1

## Data Availability

The data supporting the findings were derived from the following resources: World Values Survey, www.worldvaluessurvey.org/WVSEVSjoint2017.jsp; V‐Dem, www.v‐dem.net/data/the‐v‐dem‐dataset/; Human Development Reports, www.hdr.undp.org/data‐center/human‐development‐index#/indicies/HDI.

## Footnotes

^1^

*Electoral democracy* captures the core of polyarchy—clean, competitive elections under broad suffrage, with freedoms of association and expression and elected officials; *Liberal democracy* builds on this electoral core by embedding the rule of law, robust judicial constraints, horizontal accountability, and protections for individual and minority rights; *Participatory democracy* emphasizes citizen involvement beyond voting, reflected in a vibrant civil society, empowered local governance, and channels for direct participation; *Deliberative democracy* assesses the extent to which public decision‐making is grounded in reasoned, respectful justification oriented toward the common good rather than coercion or narrow interests; and *Egalitarian democracy* gauges the equal distribution of political power across social groups, supported by more equal access to education, health, and material resources that enable genuinely equal participation (Coppedge et al., 2024).

^2^
Only metric, but not scalar invariance was tested, because item intercept differences between groups, which are the main reasons for scalar non‐invariance, do not appear on the within‐level in our multilevel models, but are allocated to the between‐level as random parameters.
