# Supplementary material for: Context matters for the relationship between national identity and perceived democratic quality: National pride as a blind spot
Source: Br J Soc Psychol. 2026 Apr 29;65:e70084. doi: 10.1111/bjso.70084 (PMC13129263; doi:10.1111/bjso.70084)
Supplement: Supplementary file 1 — Data S1 [file BJSO-65-0-s001.docx]

**Online Supplementary Material**

1. **Descriptive statistics**
2. Descriptive statistics for country-level variables

| **Country** | **Human Development Index** | **V-Dem Electoral Democracy** | **V-Dem Liberal Democracy** | **V-Dem Participative Democracy** | **V-Dem Deliberative Democracy** | **V-Dem Egalitarian Democracy** |
| --- | --- | --- | --- | --- | --- | --- |
| Albania | 0.801 | 0.53 | 0.43 | 0.32 | 0.27 | 0.39 |
| Andorra | no data | no data | no data | no data | no data | no data |
| Azerbaijan | 0.775 | 0.19 | 0.06 | 0.06 | 0.05 | 0.12 |
| Argentina | 0.861 | 0.75 | 0.61 | 0.5 | 0.58 | 0.58 |
| Australia | 0.945 | 0.86 | 0.82 | 0.63 | 0.79 | 0.74 |
| Austria | 0.925 | 0.85 | 0.78 | 0.64 | 0.7 | 0.76 |
| Bangladesh | 0.646 | 0.26 | 0.1 | 0.12 | 0.11 | 0.1 |
| Armenia | 0.786 | 0.73 | 0.54 | 0.46 | 0.58 | 0.61 |
| Bolivia | 0.715 | 0.62 | 0.37 | 0.46 | 0.43 | 0.42 |
| Bosnia and Herzegovina | 0.788 | 0.52 | 0.35 | 0.31 | 0.36 | 0.37 |
| Brazil | 0.774 | 0.75 | 0.61 | 0.48 | 0.52 | 0.44 |
| Bulgaria | 0.829 | 0.63 | 0.53 | 0.46 | 0.49 | 0.48 |
| Myanmar | 0.609 | 0.42 | 0.26 | 0.27 | 0.34 | 0.24 |
| Belarus | 0.827 | 0.26 | 0.11 | 0.1 | 0.1 | 0.27 |
| Canada | 0.931 | 0.86 | 0.76 | 0.61 | 0.76 | 0.75 |
| Chile | 0.866 | 0.87 | 0.81 | 0.58 | 0.78 | 0.66 |
| China | 0.774 | 0.08 | 0.05 | 0.04 | 0.13 | 0.1 |
| Taiwan | no data | 0.84 | 0.72 | 0.65 | 0.72 | 0.75 |
| Colombia | 0.775 | 0.67 | 0.54 | 0.47 | 0.48 | 0.38 |
| Croatia | 0.862 | 0.74 | 0.62 | 0.52 | 0.57 | 0.58 |
| Cyprus | 0.907 | 0.84 | 0.72 | 0.57 | 0.71 | 0.74 |
| Czechia | 0.911 | 0.86 | 0.79 | 0.57 | 0.75 | 0.77 |
| Denmark | 0.951 | 0.92 | 0.89 | 0.71 | 0.88 | 0.88 |
| Ecuador | 0.767 | 0.66 | 0.47 | 0.45 | 0.5 | 0.44 |
| Ethiopia | 0.486 | 0.32 | 0.15 | 0.17 | 0.29 | 0.23 |
| Estonia | 0.897 | 0.91 | 0.86 | 0.65 | 0.84 | 0.81 |
| Finland | 0.942 | 0.87 | 0.83 | 0.61 | 0.8 | 0.8 |
| France | 0.911 | 0.88 | 0.81 | 0.61 | 0.82 | 0.78 |
| Georgia | 0.824 | 0.68 | 0.53 | 0.44 | 0.53 | 0.55 |
| Germany | 0.952 | 0.88 | 0.84 | 0.64 | 0.85 | 0.82 |
| Greece | 0.887 | 0.88 | 0.78 | 0.64 | 0.76 | 0.78 |
| Guatemala | 0.645 | 0.6 | 0.41 | 0.36 | 0.34 | 0.25 |
| Hong Kong | 0.952 | 0.32 | 0.28 | 0.12 | 0.25 | 0.29 |
| Hungary | 0.857 | 0.48 | 0.38 | 0.33 | 0.27 | 0.34 |
| Iceland | 0.962 | 0.87 | 0.78 | 0.64 | 0.75 | 0.8 |
| India | 0.685 | 0.38 | 0.28 | 0.23 | 0.27 | 0.21 |
| Indonesia | 0.715 | 0.6 | 0.47 | 0.4 | 0.51 | 0.38 |
| Iran | 0.775 | 0.17 | 0.12 | 0.04 | 0.12 | 0.16 |
| Iraq | 0.689 | 0.41 | 0.24 | 0.26 | 0.3 | 0.21 |
| Italy | 0.902 | 0.87 | 0.79 | 0.68 | 0.8 | 0.79 |
| Japan | 0.922 | 0.83 | 0.76 | 0.54 | 0.74 | 0.75 |
| Kazakhstan | 0.828 | 0.24 | 0.11 | 0.08 | 0.09 | 0.18 |
| Jordan | 0.747 | 0.26 | 0.24 | 0.12 | 0.21 | 0.23 |
| Kenya | 0.608 | 0.51 | 0.43 | 0.34 | 0.44 | 0.33 |
| South Korea | 0.924 | 0.87 | 0.8 | 0.6 | 0.79 | 0.75 |
| Kyrgyzstan | 0.7 | 0.39 | 0.26 | 0.23 | 0.24 | 0.28 |
| Lebanon | 0.774 | 0.45 | 0.28 | 0.26 | 0.29 | 0.25 |
| Latvia | 0.871 | 0.83 | 0.74 | 0.61 | 0.69 | 0.72 |
| Libya | 0.741 | 0.21 | 0.11 | 0.15 | 0.23 | 0.15 |
| Lithuania | 0.889 | 0.82 | 0.75 | 0.61 | 0.62 | 0.7 |
| Macau | no data | no data | no data | no data | no data | no data |
| Malaysia | 0.809 | 0.44 | 0.32 | 0.28 | 0.34 | 0.33 |
| Maldives | 0.745 | 0.58 | 0.42 | 0.33 | 0.45 | 0.41 |
| Mexico | 0.785 | 0.67 | 0.45 | 0.43 | 0.47 | 0.37 |
| Mongolia | 0.741 | 0.64 | 0.52 | 0.33 | 0.51 | 0.47 |
| Montenegro | 0.851 | 0.46 | 0.36 | 0.3 | 0.36 | 0.36 |
| Morocco | 0.69 | 0.26 | 0.24 | 0.16 | 0.25 | 0.21 |
| Netherlands | 0.953 | 0.86 | 0.8 | 0.58 | 0.81 | 0.77 |
| New Zealand | 0.94 | 0.89 | 0.84 | 0.69 | 0.76 | 0.79 |
| Nicaragua | 0.676 | 0.22 | 0.05 | 0.12 | 0.03 | 0.13 |
| Nigeria | 0.537 | 0.56 | 0.4 | 0.37 | 0.4 | 0.33 |
| Norway | 0.966 | 0.88 | 0.85 | 0.64 | 0.86 | 0.84 |
| Pakistan | 0.538 | 0.4 | 0.26 | 0.26 | 0.32 | 0.14 |
| Peru | 0.78 | 0.81 | 0.7 | 0.61 | 0.61 | 0.53 |
| Philippines | 0.708 | 0.45 | 0.3 | 0.31 | 0.39 | 0.2 |
| Poland | 0.882 | 0.69 | 0.54 | 0.45 | 0.47 | 0.6 |
| Portugal | 0.87 | 0.89 | 0.81 | 0.61 | 0.81 | 0.78 |
| Puerto Rico | no data | no data | no data | no data | no data | no data |
| Romania | 0.833 | 0.69 | 0.49 | 0.48 | 0.32 | 0.51 |
| Russia | 0.838 | 0.26 | 0.11 | 0.15 | 0.13 | 0.2 |
| Serbia | 0.808 | 0.38 | 0.3 | 0.25 | 0.27 | 0.31 |
| Singapore | 0.944 | 0.4 | 0.33 | 0.1 | 0.35 | 0.35 |
| Slovakia | 0.873 | 0.84 | 0.77 | 0.61 | 0.61 | 0.69 |
| Vietnam | 0.755 | 0.21 | 0.11 | 0.18 | 0.19 | 0.22 |
| Slovenia | 0.918 | 0.84 | 0.77 | 0.64 | 0.75 | 0.75 |
| Zimbabwe | 0.582 | 0.29 | 0.19 | 0.22 | 0.2 | 0.21 |
| Spain | 0.902 | 0.84 | 0.76 | 0.59 | 0.74 | 0.74 |
| Sweden | 0.949 | 0.91 | 0.88 | 0.66 | 0.87 | 0.84 |
| Switzerland | 0.96 | 0.91 | 0.87 | 0.81 | 0.88 | 0.84 |
| Tajikistan | 0.664 | 0.17 | 0.04 | 0.06 | 0.05 | 0.07 |
| Thailand | 0.796 | 0.17 | 0.11 | 0.11 | 0.03 | 0.15 |
| Tunisia | 0.74 | 0.72 | 0.63 | 0.49 | 0.65 | 0.58 |
| Turkey | 0.838 | 0.29 | 0.11 | 0.2 | 0.1 | 0.24 |
| Ukraine | 0.783 | 0.55 | 0.32 | 0.35 | 0.44 | 0.38 |
| North Macedonia | 0.805 | 0.6 | 0.42 | 0.41 | 0.45 | 0.42 |
| Egypt | 0.728 | 0.18 | 0.11 | 0.06 | 0.1 | 0.1 |
| United Kingdom (Great Britain) | 0.946 | 0.84 | 0.76 | 0.6 | 0.73 | 0.7 |
| United States | 0.931 | 0.83 | 0.75 | 0.6 | 0.64 | 0.65 |
| Uruguay | 0.852 | 0.82 | 0.75 | 0.66 | 0.7 | 0.68 |
| Uzbekistan | 0.737 | 0.22 | 0.08 | 0.06 | 0.16 | 0.16 |
| Venezuela | 0.696 | 0.22 | 0.06 | 0.16 | 0.05 | 0.12 |
| Northern Ireland | no data | no data | no data | no data | no data | no data |

1. Descriptive statistics of World Values Survey variables – Main variables

| Country | National attachment | | National pride | | Perceived democratic quality | | Votes are counted fairly | | Journalists provide fair coverage of elections | | Election officials are fair | |
| --- | --- | --- | --- | --- | --- | --- | --- | --- | --- | --- | --- | --- |
|  | Mean | SD | Mean | SD | Mean | SD | Mean | SD | Mean | SD | Mean | SD |
| Albania | 2.89 | 0.87 | 3.73 | 0.57 | 3.97 | 2.57 | 1.88 | 0.89 | 2.41 | 0.85 | 2.11 | 0.82 |
| Andorra | 3.31 | 0.79 | 3.45 | 0.69 | 6.61 | 2.03 | 3.43 | 0.82 | 3.05 | 0.84 | 3.23 | 0.85 |
| Azerbaijan | 3.13 | 0.76 | 3.65 | 0.54 | 7.33 | 1.71 | 3.16 | 0.74 | 3.00 | 0.71 | 3.10 | 0.78 |
| Argentina | 3.15 | 0.75 | 3.47 | 0.73 | 7.01 | 2.15 | 2.96 | 0.85 | 2.76 | 0.81 | 2.70 | 0.86 |
| Australia | 3.37 | 0.70 | 3.52 | 0.67 | 7.41 | 1.94 | 3.60 | 0.64 | 2.60 | 0.83 | 3.30 | 0.75 |
| Austria | 3.37 | 0.67 | 3.45 | 0.69 | 7.42 | 1.90 | 3.43 | 0.72 | 2.61 | 0.92 | 3.40 | 0.75 |
| Bangladesh | 3.28 | 0.75 | 3.81 | 0.45 | 7.23 | 2.00 | 2.87 | 0.96 | 2.66 | 0.85 | 2.81 | 0.92 |
| Armenia | 3.40 | 0.67 | 3.56 | 0.69 | 4.37 | 2.66 | 2.03 | 0.92 | 2.27 | 0.91 | 2.04 | 0.88 |
| Bolivia | 3.37 | 0.83 | 3.66 | 0.67 | 6.12 | 2.64 | 2.35 | 1.04 | 2.83 | 0.96 | 2.24 | 0.95 |
| Bosnia and Herzegovina | 3.14 | 0.84 | 2.97 | 0.85 | 3.65 | 2.40 | 1.98 | 0.88 | 2.31 | 0.87 | 2.13 | 0.87 |
| Brazil | 2.63 | 0.94 | 2.78 | 0.94 | 3.70 | 2.79 | 2.40 | 1.02 | 2.63 | 0.94 | 2.48 | 0.92 |
| Bulgaria | 3.36 | 0.62 | 3.30 | 0.79 | 4.74 | 2.50 | 2.59 | 0.90 | 2.62 | 0.84 | 2.92 | 0.80 |
| Myanmar | 3.03 | 0.85 | 3.83 | 0.44 | 6.53 | 2.62 | 2.70 | 1.16 | 2.49 | 1.17 | 2.69 | 1.12 |
| Belarus | 2.95 | 0.74 | 3.26 | 0.68 | 5.31 | 2.21 | 2.62 | 0.98 | 2.50 | 0.88 | 2.69 | 0.94 |
| Canada | 3.27 | 0.77 | 3.38 | 0.73 | 7.31 | 1.99 | 3.39 | 0.74 | 2.84 | 0.83 | 3.17 | 0.78 |
| Chile | 3.53 | 0.71 | 3.09 | 0.83 | 6.79 | 1.97 | 3.39 | 0.82 | 2.85 | 0.95 | 2.95 | 0.95 |
| China | 3.26 | 0.66 | 3.36 | 0.63 | 7.17 | 2.11 | no data | no data | no data | no data | no data | no data |
| Taiwan ROC | 3.06 | 0.59 | 2.96 | 0.73 | 7.39 | 2.15 | 3.51 | 0.74 | 2.41 | 0.95 | 2.83 | 0.95 |
| Colombia | 3.16 | 0.83 | 3.74 | 0.62 | 5.24 | 3.18 | 2.19 | 1.05 | 2.98 | 1.11 | 2.28 | 1.06 |
| Croatia | 3.22 | 0.75 | 3.14 | 0.84 | 4.19 | 2.33 | 2.51 | 0.89 | 2.31 | 0.84 | 2.52 | 0.85 |
| Cyprus | 3.34 | 0.84 | 3.32 | 0.86 | 6.12 | 2.39 | 2.98 | 0.95 | 2.48 | 0.81 | 2.80 | 0.92 |
| Czechia | 3.34 | 0.69 | 3.10 | 0.76 | 6.04 | 2.26 | 3.18 | 0.86 | 2.69 | 0.89 | 3.10 | 0.87 |
| Denmark | 3.56 | 0.59 | 3.41 | 0.65 | 8.54 | 1.50 | 3.83 | 0.47 | 3.00 | 0.74 | 3.66 | 0.62 |
| Ecuador | 3.16 | 0.72 | 3.82 | 0.52 | 6.26 | 2.61 | 2.42 | 0.97 | 2.94 | 0.87 | 2.34 | 0.92 |
| Ethiopia | 2.92 | 0.91 | 3.82 | 0.51 | 5.43 | 2.99 | 2.23 | 1.04 | 2.08 | 1.01 | 2.08 | 1.01 |
| Estonia | 3.47 | 0.60 | 3.40 | 0.63 | 6.33 | 2.15 | 3.26 | 0.80 | 2.69 | 0.78 | 3.21 | 0.81 |
| Finland | 3.58 | 0.56 | 3.65 | 0.56 | 7.25 | 2.03 | 3.69 | 0.79 | 2.77 | 0.81 | 3.71 | 0.57 |
| France | 3.42 | 0.71 | 3.41 | 0.71 | 6.47 | 2.24 | 3.29 | 0.80 | 2.49 | 0.84 | 2.84 | 0.81 |
| Georgia | 3.19 | 0.72 | 3.61 | 0.63 | 4.82 | 2.66 | 2.20 | 0.99 | 2.51 | 0.86 | 2.28 | 0.93 |
| Germany | 3.34 | 0.64 | 3.04 | 0.85 | 7.32 | 2.03 | 3.63 | 0.64 | 2.89 | 0.80 | 3.61 | 0.61 |
| Greece | 3.58 | 0.61 | 3.69 | 0.58 | 5.40 | 2.34 | 3.12 | 0.85 | 2.01 | 0.83 | 2.71 | 0.94 |
| Guatemala | 2.95 | 0.82 | 3.56 | 0.68 | 4.92 | 2.72 | 2.15 | 0.96 | 2.76 | 0.95 | 2.24 | 0.97 |
| Hong Kong SAR | 3.06 | 0.62 | 2.77 | 0.78 | 5.76 | 2.01 | 3.26 | 0.72 | 2.83 | 0.70 | 2.92 | 0.74 |
| Hungary | 3.28 | 0.70 | 3.35 | 0.74 | 5.35 | 2.74 | 3.09 | 0.94 | 2.16 | 0.92 | 3.02 | 0.93 |
| Iceland | 3.50 | 0.60 | 3.57 | 0.61 | 6.63 | 2.11 | 3.84 | 0.41 | 2.79 | 0.72 | 2.75 | 0.62 |
| India | 3.57 | 0.77 | 3.70 | 0.56 | 6.85 | 2.50 | 3.11 | 0.90 | 2.71 | 0.99 | 3.08 | 0.91 |
| Indonesia | 3.01 | 0.90 | 3.78 | 0.46 | 7.33 | 2.37 | 3.24 | 0.88 | 2.60 | 1.00 | 2.91 | 0.91 |
| Iran | 3.77 | 0.55 | 3.78 | 0.58 | 6.49 | 2.97 | 3.10 | 1.06 | 2.53 | 1.11 | 2.87 | 1.00 |
| Iraq | 3.56 | 0.88 | 3.46 | 0.88 | 4.67 | 2.61 | 1.93 | 1.00 | 2.60 | 1.08 | 2.05 | 1.05 |
| Italy | 3.05 | 0.76 | 3.25 | 0.72 | 6.04 | 2.25 | 2.93 | 0.84 | 2.51 | 0.83 | 3.23 | 0.76 |
| Japan | 3.25 | 0.68 | 3.16 | 0.73 | 7.13 | 1.83 | 3.34 | 0.90 | 2.72 | 0.81 | 3.16 | 0.90 |
| Kazakhstan | 3.23 | 0.77 | 3.41 | 0.67 | 6.48 | 2.13 | 2.85 | 0.88 | 2.66 | 0.91 | 2.76 | 0.90 |
| Jordan | 3.93 | 0.32 | 3.88 | 0.42 | 6.65 | 2.34 | 2.33 | 1.01 | 2.48 | 0.99 | 2.37 | 1.00 |
| Kenya | 3.15 | 0.83 | 3.37 | 0.82 | 5.49 | 2.86 | 2.23 | 0.96 | 2.83 | 0.93 | 2.26 | 0.99 |
| South Korea | 3.24 | 0.67 | 2.91 | 0.55 | 6.88 | 1.19 | 3.12 | 0.60 | 2.66 | 0.67 | 2.82 | 0.67 |
| Kyrgyzstan | 3.57 | 0.63 | 3.72 | 0.58 | 6.35 | 2.92 | 2.33 | 1.02 | 2.67 | 0.94 | 2.26 | 0.98 |
| Lebanon | 3.70 | 0.66 | 3.42 | 0.73 | 4.32 | 2.21 | 2.46 | 0.93 | 2.58 | 0.80 | 2.34 | 0.85 |
| Latvia | 3.27 | 0.63 | 3.18 | 0.70 | 5.62 | 2.20 | 3.23 | 0.82 | 2.91 | 0.76 | 3.20 | 0.73 |
| Libya | 3.93 | 0.33 | 3.80 | 0.47 | 3.84 | 2.83 | 2.66 | 1.17 | 2.45 | 1.14 | 2.40 | 1.13 |
| Lithuania | 3.14 | 0.61 | 3.09 | 0.72 | 5.83 | 2.05 | 2.86 | 0.84 | 2.39 | 0.83 | 2.40 | 0.92 |
| Macau SAR | 2.89 | 0.76 | 2.96 | 0.67 | 5.76 | 1.84 | 3.00 | 0.81 | 2.81 | 0.77 | 2.84 | 0.75 |
| Malaysia | 3.00 | 0.84 | 3.05 | 0.87 | 6.06 | 2.41 | 2.64 | 0.93 | 2.48 | 0.88 | 2.53 | 0.90 |
| Maldives | 3.24 | 0.79 | 3.59 | 0.71 | 4.75 | 2.63 | 2.76 | 0.89 | 2.74 | 0.84 | 2.61 | 0.88 |
| Mexico | 3.42 | 0.82 | 3.62 | 0.70 | 5.22 | 2.88 | 2.11 | 1.02 | 2.74 | 1.10 | 2.10 | 1.02 |
| Mongolia | 3.07 | 0.87 | 3.13 | 0.84 | 6.24 | 1.93 | 2.74 | 0.87 | 2.63 | 0.85 | 2.63 | 0.88 |
| Montenegro | 3.29 | 0.67 | 3.25 | 0.62 | 5.18 | 2.46 | 2.43 | 1.06 | 2.25 | 0.99 | 2.24 | 1.00 |
| Morocco | 3.47 | 0.87 | 3.32 | 0.67 | 5.50 | 2.21 | 3.01 | 0.91 | 2.78 | 0.85 | 2.79 | 0.86 |
| Netherlands | 3.09 | 0.69 | 3.08 | 0.74 | 7.22 | 1.91 | 3.63 | 0.64 | 2.88 | 0.79 | 3.08 | 0.83 |
| New Zealand | 3.35 | 0.69 | 3.65 | 0.54 | 7.50 | 1.99 | 3.81 | 0.45 | 2.85 | 0.79 | 3.55 | 0.61 |
| Nicaragua | 3.00 | 0.83 | 3.81 | 0.59 | 4.83 | 3.47 | 2.31 | 1.17 | 2.45 | 1.06 | 2.23 | 1.09 |
| Nigeria | 3.46 | 0.66 | 3.57 | 0.75 | 5.57 | 2.83 | 2.54 | 0.92 | 2.80 | 0.85 | 2.49 | 0.90 |
| Norway | 3.81 | 0.44 | 3.63 | 0.58 | 7.93 | 1.68 | 3.87 | 0.40 | 2.93 | 0.77 | 3.57 | 0.65 |
| Pakistan | 3.67 | 0.70 | 3.81 | 0.52 | 6.74 | 2.85 | 2.87 | 1.04 | 2.74 | 1.06 | 2.77 | 1.02 |
| Peru | 3.47 | 0.76 | 3.66 | 0.62 | 5.45 | 2.68 | 2.10 | 0.79 | 2.28 | 0.80 | 1.91 | 0.72 |
| Philippines | 3.30 | 0.65 | 3.87 | 0.41 | 6.92 | 2.41 | 3.01 | 0.85 | 2.96 | 0.81 | 2.90 | 0.80 |
| Poland | 3.46 | 0.61 | 3.63 | 0.55 | 5.63 | 2.74 | 3.15 | 0.76 | 2.55 | 0.79 | 3.08 | 0.70 |
| Portugal | 3.36 | 0.66 | 3.68 | 0.53 | 6.79 | 1.91 | 3.60 | 0.67 | 2.97 | 0.82 | 3.33 | 0.84 |
| Puerto Rico | 3.45 | 0.72 | 3.85 | 0.48 | 5.46 | 3.01 | 2.14 | 1.05 | 2.83 | 1.07 | 2.17 | 1.03 |
| Romania | 3.39 | 0.67 | 3.38 | 0.71 | 5.43 | 2.83 | 2.48 | 1.01 | 2.39 | 0.86 | 2.44 | 0.95 |
| Russia | 3.06 | 0.83 | 3.37 | 0.69 | 5.62 | 2.22 | 2.66 | 0.91 | 2.55 | 0.87 | 2.68 | 0.86 |
| Serbia | 3.07 | 0.77 | 3.18 | 0.76 | 4.91 | 2.40 | 2.57 | 0.97 | 2.43 | 0.97 | 2.28 | 0.92 |
| Singapore | 3.13 | 0.61 | 3.44 | 0.67 | 6.75 | 1.81 | 3.55 | 0.65 | 2.80 | 0.84 | 3.41 | 0.66 |
| Slovakia | 3.41 | 0.60 | 3.23 | 0.69 | 5.66 | 2.37 | 3.17 | 0.83 | 2.65 | 0.89 | 2.99 | 0.91 |
| Vietnam | 3.23 | 0.65 | 3.54 | 0.60 | 7.81 | 2.04 | 3.03 | 0.83 | 2.72 | 0.95 | 2.75 | 0.89 |
| Slovenia | 3.36 | 0.60 | 3.46 | 0.68 | 5.38 | 2.18 | 3.40 | 0.77 | 2.73 | 0.82 | 3.34 | 0.72 |
| Zimbabwe | 3.44 | 0.79 | 3.40 | 0.96 | 3.95 | 3.22 | 2.44 | 1.22 | 2.61 | 1.19 | 2.50 | 1.18 |
| Spain | 3.23 | 0.78 | 3.24 | 0.82 | 6.69 | 2.40 | 3.56 | 0.75 | 2.66 | 0.94 | 3.22 | 0.93 |
| Sweden | 3.34 | 0.63 | 3.47 | 0.67 | 7.90 | 1.77 | 3.84 | 0.45 | 2.99 | 0.77 | 3.68 | 0.57 |
| Switzerland | 3.52 | 0.62 | 3.30 | 0.75 | 7.83 | 1.67 | 3.67 | 0.57 | 2.81 | 0.73 | 3.20 | 0.78 |
| Tajikistan | 3.53 | 0.74 | 3.74 | 0.48 | 7.98 | 1.86 | 3.49 | 0.77 | 2.97 | 0.96 | 3.29 | 0.90 |
| Thailand | 2.52 | 0.96 | 3.68 | 0.60 | 5.90 | 2.28 | 3.07 | 0.84 | 2.71 | 0.90 | 3.05 | 0.86 |
| Tunisia | 3.66 | 0.63 | 3.50 | 0.74 | 4.84 | 2.47 | 2.65 | 1.09 | 2.67 | 0.96 | 2.57 | 1.03 |
| Turkey | 3.24 | 0.76 | 3.56 | 0.66 | 6.27 | 2.29 | 3.00 | 0.96 | 2.54 | 0.91 | 2.95 | 0.94 |
| Ukraine | 3.01 | 0.79 | 3.17 | 0.77 | 4.99 | 2.44 | 2.70 | 0.83 | 2.53 | 0.77 | 2.73 | 0.83 |
| North Macedonia | 2.93 | 0.90 | 3.02 | 0.95 | 4.05 | 2.56 | 2.39 | 0.94 | 2.37 | 0.83 | 2.49 | 0.86 |
| Egypt | 3.89 | 0.39 | 3.42 | 0.62 | 4.42 | 2.51 | 2.31 | 1.00 | 2.01 | 0.90 | 2.35 | 0.98 |
| Great Britain | 2.95 | 0.80 | 3.25 | 0.83 | 6.56 | 2.24 | 3.51 | 0.70 | 2.42 | 0.85 | 3.23 | 0.79 |
| United States | 2.97 | 0.83 | 3.16 | 0.88 | 6.05 | 2.23 | 3.11 | 0.87 | 2.33 | 0.94 | 2.86 | 0.88 |
| Uruguay | 3.57 | 0.65 | 3.63 | 0.61 | 7.76 | 2.47 | 3.48 | 0.84 | 2.64 | 1.07 | 3.11 | 0.99 |
| Uzbekistan | 3.53 | 0.70 | 3.82 | 0.50 | 7.52 | 2.27 | 3.33 | 0.80 | 2.91 | 0.96 | 2.94 | 0.96 |
| Venezuela | 3.41 | 0.74 | 3.70 | 0.66 | 4.96 | 2.82 | 2.15 | 1.06 | 2.59 | 0.96 | 2.13 | 1.02 |
| Northern Ireland | 2.56 | 0.92 | 3.15 | 0.86 | 5.88 | 2.59 | 3.44 | 0.68 | 2.64 | 0.83 | 3.28 | 0.76 |

3. Descriptive statistics of World Values Survey variables – Control variables

| Country | Confidence - Government | | Gender | | Age | | Education | | Left-right position | | Religion | |
| --- | --- | --- | --- | --- | --- | --- | --- | --- | --- | --- | --- | --- |
|  | Mean | SD | Mean | SD | Mean | SD | Mean | SD | Mean | SD | Mean | SD |
| Albania | 1.61 | 0.81 | 1.63 | 0.48 | 45.58 | 16.34 | 1.70 | 0.71 | 5.40 | 3.52 | 2.78 | 0.47 |
| Andorra | 2.44 | 0.80 | 1.49 | 0.50 | 46.74 | 15.94 | 2.12 | 0.83 | 4.86 | 1.95 | 2.30 | 0.72 |
| Azerbaijan | 3.32 | 0.64 | 1.51 | 0.50 | 40.68 | 14.68 | 2.06 | 0.48 | 6.37 | 2.24 | 2.90 | 0.33 |
| Argentina | 2.06 | 0.88 | 1.52 | 0.50 | 42.51 | 17.34 | 1.75 | 0.70 | 5.90 | 2.15 | 2.67 | 0.59 |
| Australia | 2.18 | 0.75 | 1.61 | 0.49 | 54.09 | 17.00 | 2.36 | 0.64 | 5.29 | 2.10 | 2.19 | 0.74 |
| Austria | 2.29 | 0.78 | 1.55 | 0.50 | 50.84 | 17.24 | 2.03 | 0.56 | 5.17 | 1.87 | 2.57 | 0.60 |
| Bangladesh | 3.11 | 0.80 | 1.51 | 0.50 | 36.59 | 12.99 | 1.30 | 0.62 | 7.17 | 2.14 | 2.98 | 0.15 |
| Armenia | 2.03 | 0.93 | 1.61 | 0.49 | 46.48 | 17.34 | 2.35 | 0.64 | 4.96 | 2.67 | 2.86 | 0.40 |
| Bolivia | 2.06 | 0.88 | 1.50 | 0.50 | 38.33 | 15.90 | 2.00 | 0.83 | 5.36 | 2.40 | 2.84 | 0.40 |
| Bosnia and Herzegovina | 1.75 | 0.82 | 1.56 | 0.50 | 46.28 | 17.67 | 1.95 | 0.62 | 5.35 | 2.56 | 2.83 | 0.43 |
| Brazil | 1.71 | 0.88 | 1.55 | 0.50 | 43.52 | 17.26 | 1.73 | 0.72 | 5.41 | 2.78 | 2.73 | 0.48 |
| Bulgaria | 1.98 | 0.78 | 1.60 | 0.49 | 53.02 | 17.16 | 2.05 | 0.66 | 5.80 | 2.74 | 2.62 | 0.55 |
| Myanmar | 3.10 | 0.86 | 1.50 | 0.50 | 40.43 | 14.30 | 1.48 | 0.69 | no data | no data | 2.95 | 0.23 |
| Belarus | 2.54 | 0.88 | 1.57 | 0.50 | 46.81 | 16.44 | 2.59 | 0.63 | 5.34 | 1.82 | 2.29 | 0.61 |
| Canada | 2.41 | 0.81 | 1.49 | 0.50 | 46.54 | 16.82 | 2.50 | 0.60 | 5.25 | 2.04 | 2.15 | 0.73 |
| Chile | 2.18 | 0.85 | 1.53 | 0.50 | 45.25 | 15.55 | 2.17 | 0.61 | 5.08 | 1.85 | 2.40 | 0.63 |
| China | 3.44 | 0.60 | 1.55 | 0.50 | 44.58 | 14.50 | 1.68 | 0.82 | no data | no data | 1.81 | 0.69 |
| Taiwan ROC | 2.45 | 0.80 | 1.51 | 0.50 | 48.27 | 16.77 | 2.31 | 0.79 | 3.96 | 1.85 | 2.22 | 0.76 |
| Colombia | 1.88 | 0.78 | 1.50 | 0.50 | 38.84 | 15.84 | 1.84 | 0.76 | 6.08 | 2.93 | 2.72 | 0.50 |
| Croatia | 1.61 | 0.66 | 1.58 | 0.49 | 48.89 | 17.69 | 2.17 | 0.64 | 5.34 | 2.62 | 2.75 | 0.57 |
| Cyprus | 2.29 | 0.94 | 1.52 | 0.50 | 43.52 | 16.01 | 2.27 | 0.81 | 5.47 | 2.59 | 2.71 | 0.48 |
| Czechia | 2.00 | 0.76 | 1.58 | 0.49 | 50.40 | 17.20 | 1.96 | 0.64 | 5.71 | 2.23 | 2.16 | 0.69 |
| Denmark | 2.31 | 0.76 | 1.52 | 0.50 | 51.82 | 17.64 | 2.32 | 0.76 | 5.25 | 2.23 | 2.51 | 0.66 |
| Ecuador | 2.09 | 0.88 | 1.52 | 0.50 | 39.49 | 15.61 | 1.94 | 0.75 | 5.77 | 2.60 | 2.75 | 0.46 |
| Ethiopia | 2.81 | 1.01 | 1.49 | 0.50 | 31.93 | 11.63 | 1.49 | 0.74 | 6.46 | 3.44 | 2.93 | 0.26 |
| Estonia | 2.39 | 0.73 | 1.63 | 0.48 | 54.62 | 18.38 | 2.16 | 0.66 | 5.63 | 2.01 | 2.31 | 0.60 |
| Finland | 2.37 | 0.75 | 1.52 | 0.50 | 53.76 | 18.20 | 2.27 | 0.73 | 5.88 | 2.22 | 2.46 | 0.64 |
| France | 2.03 | 0.84 | 1.55 | 0.50 | 51.93 | 18.34 | 2.07 | 0.74 | 5.17 | 2.11 | 2.17 | 0.78 |
| Georgia | 2.19 | 0.97 | 1.63 | 0.48 | 50.89 | 17.59 | 2.17 | 0.66 | 6.30 | 2.56 | 2.95 | 0.26 |
| Germany | 2.30 | 0.75 | 1.50 | 0.50 | 50.80 | 17.72 | 2.24 | 0.64 | 4.84 | 1.74 | 2.40 | 0.69 |
| Greece | 1.65 | 0.74 | 1.53 | 0.50 | 50.79 | 17.56 | 1.92 | 0.75 | 5.38 | 2.06 | 2.81 | 0.47 |
| Guatemala | 1.68 | 0.67 | 1.53 | 0.50 | 33.49 | 14.34 | 2.31 | 0.77 | 5.79 | 2.47 | 2.63 | 0.53 |
| Hong Kong SAR | 2.50 | 0.84 | 1.54 | 0.50 | 47.16 | 15.80 | 2.19 | 0.75 | 5.69 | 1.54 | 1.63 | 0.73 |
| Hungary | 2.20 | 0.99 | 1.57 | 0.50 | 49.79 | 18.64 | 2.10 | 0.64 | 6.04 | 2.41 | 2.48 | 0.63 |
| Iceland | 2.15 | 0.78 | 1.52 | 0.50 | 48.83 | 17.29 | 2.20 | 0.79 | 5.24 | 2.21 | 2.37 | 0.73 |
| India | 2.79 | 1.01 | 1.43 | 0.50 | 35.80 | 16.30 | 1.91 | 0.74 | 6.35 | 2.40 | 2.77 | 0.48 |
| Indonesia | 3.15 | 0.80 | 1.55 | 0.50 | 40.02 | 13.52 | 1.53 | 0.67 | 6.37 | 2.72 | 2.93 | 0.26 |
| Iran | 2.42 | 1.10 | 1.49 | 0.50 | 39.37 | 14.56 | 2.12 | 0.78 | no data | no data | 2.83 | 0.41 |
| Iraq | 1.73 | 0.94 | 1.49 | 0.50 | 36.60 | 13.40 | 1.74 | 0.84 | no data | no data | 2.72 | 0.55 |
| Italy | 2.01 | 0.78 | 1.50 | 0.50 | 51.25 | 17.92 | 1.77 | 0.71 | 5.60 | 2.35 | 2.70 | 0.59 |
| Japan | 2.36 | 0.72 | 1.56 | 0.50 | 54.69 | 17.80 | 2.49 | 0.61 | 5.75 | 2.00 | 1.95 | 0.61 |
| Kazakhstan | 2.89 | 0.80 | 1.55 | 0.50 | 41.24 | 14.21 | 2.51 | 0.61 | no data | no data | 2.87 | 0.40 |
| Jordan | 2.19 | 1.14 | 1.50 | 0.50 | 43.31 | 14.85 | 1.90 | 0.77 | no data | no data | 2.79 | 0.41 |
| Kenya | 2.43 | 1.02 | 1.49 | 0.50 | 30.74 | 10.06 | 1.93 | 0.74 | 5.51 | 2.69 | 2.92 | 0.29 |
| South Korea | 2.48 | 0.70 | 1.51 | 0.50 | 45.62 | 15.01 | 2.36 | 0.67 | 5.27 | 1.76 | 1.61 | 0.75 |
| Kyrgyzstan | 2.48 | 0.91 | 1.62 | 0.49 | 41.29 | 15.16 | 2.39 | 0.61 | no data | no data | 2.75 | 0.48 |
| Lebanon | 1.91 | 0.77 | 1.50 | 0.50 | 40.82 | 15.40 | 2.04 | 0.83 | no data | no data | 2.56 | 0.54 |
| Latvia | 2.06 | 0.81 | 1.65 | 0.48 | 51.11 | 17.03 | 2.23 | 0.67 | 6.00 | 2.06 | 2.58 | 0.61 |
| Libya | 2.08 | 0.95 | 1.48 | 0.50 | 40.21 | 12.70 | 2.45 | 0.66 | 5.75 | 2.48 | 2.75 | 0.44 |
| Lithuania | 2.34 | 0.67 | 1.61 | 0.49 | 49.84 | 18.01 | 2.20 | 0.74 | 5.88 | 2.04 | 2.83 | 0.43 |
| Macau SAR | 2.78 | 0.81 | 1.56 | 0.50 | 40.69 | 16.74 | 2.11 | 0.84 | 5.15 | 1.37 | 1.55 | 0.73 |
| Malaysia | 2.48 | 0.87 | 1.50 | 0.50 | 38.33 | 13.21 | 1.77 | 0.87 | 5.90 | 1.82 | 2.77 | 0.48 |
| Maldives | 2.03 | 0.98 | 1.53 | 0.50 | 37.65 | 14.09 | 1.60 | 0.87 | 5.97 | 2.59 | 2.85 | 0.35 |
| Mexico | 1.68 | 0.87 | 1.50 | 0.50 | 43.33 | 16.68 | 1.83 | 0.67 | 5.65 | 2.75 | 2.70 | 0.47 |
| Mongolia | 2.30 | 0.91 | 1.52 | 0.50 | 38.79 | 14.25 | 2.43 | 0.67 | 6.18 | 2.09 | 2.25 | 0.66 |
| Montenegro | 2.21 | 0.88 | 1.50 | 0.50 | 47.25 | 16.85 | 2.13 | 0.60 | 5.03 | 2.61 | 2.87 | 0.41 |
| Morocco | 1.98 | 0.73 | 1.50 | 0.50 | 37.22 | 13.53 | 1.72 | 0.82 | 5.79 | 2.33 | 2.64 | 0.48 |
| Netherlands | 2.30 | 0.73 | 1.54 | 0.50 | 53.14 | 16.80 | 2.21 | 0.81 | 5.48 | 2.13 | 2.28 | 0.69 |
| New Zealand | 2.55 | 0.76 | 1.57 | 0.49 | 57.68 | 16.20 | 2.42 | 0.75 | 5.47 | 2.31 | 2.21 | 0.67 |
| Nicaragua | 2.06 | 1.07 | 1.51 | 0.50 | 35.13 | 14.08 | 1.67 | 0.81 | 5.59 | 3.19 | 2.67 | 0.50 |
| Nigeria | 2.31 | 0.99 | 1.49 | 0.50 | 32.54 | 12.03 | 1.68 | 0.64 | 5.98 | 2.78 | 2.94 | 0.25 |
| Norway | 2.63 | 0.69 | 1.51 | 0.50 | 48.32 | 17.34 | 2.29 | 0.75 | 5.36 | 2.16 | 2.29 | 0.63 |
| Pakistan | 2.71 | 1.08 | 1.48 | 0.50 | 35.64 | 11.38 | 1.56 | 0.67 | no data | no data | 2.95 | 0.25 |
| Peru | 1.55 | 0.74 | 1.50 | 0.50 | 40.15 | 15.51 | 1.96 | 0.71 | 6.10 | 2.27 | 2.80 | 0.42 |
| Philippines | 3.12 | 0.76 | 1.50 | 0.50 | 43.69 | 16.02 | 1.45 | 0.75 | 6.66 | 2.54 | 2.84 | 0.37 |
| Poland | 1.98 | 0.88 | 1.54 | 0.50 | 49.80 | 17.55 | 1.94 | 0.78 | 6.30 | 2.33 | 2.83 | 0.46 |
| Portugal | 2.15 | 0.80 | 1.59 | 0.49 | 55.41 | 18.46 | 1.52 | 0.74 | 5.02 | 1.87 | 2.70 | 0.58 |
| Puerto Rico | 1.69 | 0.82 | 1.61 | 0.49 | 49.67 | 18.20 | 2.42 | 0.74 | 5.34 | 3.33 | 2.70 | 0.50 |
| Romania | 1.81 | 0.86 | 1.57 | 0.50 | 48.71 | 17.85 | 1.89 | 0.68 | 5.69 | 2.74 | 2.81 | 0.41 |
| Russia | 2.52 | 0.92 | 1.58 | 0.49 | 45.51 | 17.17 | 2.52 | 0.71 | 5.96 | 2.03 | 2.74 | 0.58 |
| Serbia | 1.96 | 0.86 | 1.53 | 0.50 | 45.93 | 16.75 | 2.21 | 0.67 | 5.12 | 2.23 | 2.73 | 0.54 |
| Singapore | 3.01 | 0.68 | 1.54 | 0.50 | 47.75 | 16.17 | 2.32 | 0.77 | 5.08 | 2.02 | 2.47 | 0.66 |
| Slovakia | 2.01 | 0.85 | 1.58 | 0.49 | 51.54 | 16.52 | 1.94 | 0.63 | 5.55 | 2.08 | 2.67 | 0.59 |
| Vietnam | 3.30 | 0.60 | 1.55 | 0.50 | 37.89 | 12.72 | 1.90 | 0.74 | no data | no data | 2.19 | 0.63 |
| Slovenia | 1.90 | 0.69 | 1.56 | 0.50 | 50.57 | 18.10 | 2.14 | 0.67 | 4.78 | 2.00 | 2.54 | 0.73 |
| Zimbabwe | 2.47 | 1.04 | 1.51 | 0.50 | 39.10 | 16.22 | 1.51 | 0.63 | 5.43 | 3.28 | 2.96 | 0.20 |
| Spain | 1.88 | 0.86 | 1.55 | 0.50 | 50.20 | 17.44 | 1.68 | 0.81 | 4.87 | 2.33 | 2.37 | 0.71 |
| Sweden | 2.50 | 0.73 | 1.52 | 0.50 | 51.75 | 17.94 | 2.33 | 0.69 | 5.48 | 2.09 | 2.12 | 0.68 |
| Switzerland | 2.74 | 0.69 | 1.53 | 0.50 | 48.76 | 17.63 | 2.27 | 0.71 | 5.29 | 2.02 | 2.38 | 0.67 |
| Tajikistan | 3.43 | 0.71 | 1.51 | 0.50 | 41.06 | 15.30 | 2.30 | 0.66 | 7.55 | 2.26 | 2.41 | 0.50 |
| Thailand | 2.60 | 0.84 | 1.53 | 0.50 | 46.22 | 13.17 | 1.43 | 0.72 | 5.99 | 1.87 | 2.23 | 0.48 |
| Tunisia | 1.60 | 0.81 | 1.54 | 0.50 | 43.15 | 15.54 | 1.67 | 0.70 | 6.03 | 2.34 | 2.60 | 0.53 |
| Turkey | 2.86 | 0.87 | 1.50 | 0.50 | 38.83 | 12.65 | 1.59 | 0.77 | 6.29 | 2.57 | 2.71 | 0.48 |
| Ukraine | 1.83 | 0.79 | 1.61 | 0.49 | 47.14 | 16.45 | 2.49 | 0.68 | 5.79 | 2.19 | 2.67 | 0.58 |
| North Macedonia | 1.86 | 0.92 | 1.50 | 0.50 | 43.83 | 16.72 | 2.18 | 0.70 | 5.39 | 2.68 | 2.78 | 0.51 |
| Egypt | no data | no data | 1.48 | 0.50 | 39.70 | 13.45 | 1.80 | 0.76 | no data | no data | 2.74 | 0.44 |
| Great Britain | 2.03 | 0.79 | 1.57 | 0.50 | 52.21 | 17.77 | 2.18 | 0.89 | 5.10 | 1.95 | 2.17 | 0.70 |
| United States | 2.08 | 0.91 | 1.46 | 0.50 | 43.39 | 16.24 | 2.50 | 0.53 | 5.22 | 2.52 | 2.46 | 0.66 |
| Uruguay | 2.50 | 1.10 | 1.68 | 0.47 | 49.76 | 17.94 | 1.90 | 0.84 | 5.22 | 3.12 | 2.41 | 0.71 |
| Uzbekistan | 3.29 | 0.79 | 1.49 | 0.50 | 35.90 | 12.89 | 2.44 | 0.58 | no data | no data | 2.45 | 0.54 |
| Venezuela | 1.69 | 0.85 | 1.52 | 0.50 | 38.30 | 15.24 | 1.87 | 0.79 | 6.29 | 2.67 | 2.55 | 0.53 |
| Northern Ireland | 1.84 | 0.74 | 1.50 | 0.50 | 51.97 | 17.01 | 1.98 | 0.89 | 5.12 | 1.99 | 2.42 | 0.65 |

1. **Summary of the alignment optimization measurement invariance test for the latent variable of perceived electoral quality**

Average R^2^ = 0.445

***Countries flagged as invariant on the 3 indicator items of perceived electoral quality***

1. *Item: “Votes are counted fairly.”*

| **Countries flagged as invariant** | |
| --- | --- |
| Country Code^[[1]](#footnote-1)^ | Country |
| 8 | Albania |
| 20 | Andorra |
| 31 | Azerbaijan |
| 32 | Argentina |
| 36 | Australia |
| 40 | Austria |
| 50 | Bangladesh |
| 51 | Armenia |
| 68 | Bolivia |
| 70 | Bosnia and Herzegovina |
| 76 | Brazil |
| 104 | Myanmar |
| 170 | Colombia |
| 191 | Croatia |
| 196 | Cyprus |
| 203 | Czechia |
| 208 | Denmark |
| 218 | Ecuador |
| 246 | Finland |
| 250 | France |
| 268 | Georgia |
| 276 | Germany |
| 300 | Greece |
| 320 | Guatemala |
| 344 | Hong Kong |
| 348 | Hungary |
| 356 | India |
| 360 | Indonesia |
| 364 | Iran |
| 392 | Japan |
| 398 | Kazakhstan |
| 404 | Kenya |
| 410 | South Korea |
| 417 | Kyrgyzstan |
| 422 | Lebanon |
| 434 | Libya |
| 446 | Macao |
| 458 | Malaysia |
| 484 | Mexico |
| 499 | Montenegro |
| 504 | Morocco |
| 554 | New Zealand |
| 558 | Nicaragua |
| 566 | Nigeria |
| 604 | Peru |
| 616 | Poland |
| 630 | Puerto Rico |
| 688 | Serbia |
| 702 | Singapore |
| 703 | Slovakia |
| 716 | Zimbabwe |
| 724 | Spain |
| 756 | Switzerland |
| 762 | Tajikistan |
| 764 | Thailand |
| 788 | Tunisia |
| 792 | Turkey |
| 807 | North Macedonia |
| 826 | United Kingdom (Great Britain) |
| 858 | Uruguay |
| 909 | United Kingdom (Northern Ireland) |

Weighted Average Factor Score Across Invariant Groups:

0.663

| **Unstandardized factor weights for the invariant countries, and their differences from the average** | | | | |
| --- | --- | --- | --- | --- |
| Country Code | Value | Difference | SE | p |
| 8 | 0.656 | -0.007 | 0.054 | 0.904 |
| 20 | 0.656 | -0.007 | 0.016 | 0.650 |
| 31 | 0.674 | 0.012 | 0.016 | 0.473 |
| 32 | 0.661 | -0.002 | 0.010 | 0.837 |
| 36 | 0.680 | 0.018 | 0.017 | 0.309 |
| 40 | 0.723 | 0.060 | 0.022 | 0.007 |
| 50 | 0.683 | 0.020 | 0.021 | 0.336 |
| 51 | 0.691 | 0.028 | 0.024 | 0.244 |
| 68 | 0.766 | 0.103 | 0.037 | 0.005 |
| 70 | 0.682 | 0.019 | 0.023 | 0.394 |
| 76 | 0.663 | 0.001 | 0.018 | 0.969 |
| 104 | 0.604 | -0.059 | 0.031 | 0.061 |
| 170 | 0.666 | 0.004 | 0.013 | 0.781 |
| 191 | 0.705 | 0.042 | 0.016 | 0.010 |
| 196 | 0.724 | 0.061 | 0.023 | 0.008 |
| 422 | 0.711 | 0.049 | 0.023 | 0.036 |
| 368 | 0.531 | -0.131 | 0.057 | 0.022 |
| 400 | 0.605 | -0.058 | 0.035 | 0.098 |
| 203 | 0.699 | 0.036 | 0.013 | 0.007 |
| 208 | 0.530 | -0.132 | 0.043 | 0.002 |
| 218 | 0.634 | -0.029 | 0.063 | 0.645 |
| 246 | 0.478 | -0.185 | 0.072 | 0.011 |
| 250 | 0.552 | -0.110 | 0.036 | 0.003 |
| 268 | 0.682 | 0.019 | 0.018 | 0.298 |
| 276 | 0.710 | 0.047 | 0.017 | 0.006 |
| 300 | 0.602 | -0.061 | 0.043 | 0.160 |
| 320 | 0.677 | 0.014 | 0.020 | 0.482 |
| 344 | 0.646 | -0.017 | 0.025 | 0.510 |
| 348 | 0.740 | 0.077 | 0.040 | 0.053 |
| 356 | 0.742 | 0.080 | 0.030 | 0.007 |
| 360 | 0.738 | 0.075 | 0.030 | 0.012 |
| 364 | 0.718 | 0.055 | 0.030 | 0.064 |
| 392 | 0.702 | 0.040 | 0.015 | 0.008 |
| 398 | 0.645 | -0.018 | 0.028 | 0.522 |
| 404 | 0.779 | 0.116 | 0.040 | 0.003 |
| 410 | 0.610 | -0.053 | 0.044 | 0.234 |
| 417 | 0.550 | -0.112 | 0.053 | 0.033 |
| 434 | 0.676 | 0.013 | 0.032 | 0.677 |
| 446 | 0.626 | -0.037 | 0.020 | 0.062 |
| 458 | 0.702 | 0.039 | 0.018 | 0.027 |
| 484 | 0.667 | 0.005 | 0.028 | 0.869 |
| 499 | 0.659 | -0.003 | 0.027 | 0.908 |
| 504 | 0.687 | 0.025 | 0.021 | 0.232 |
| 554 | 0.549 | -0.114 | 0.070 | 0.102 |
| 558 | 0.689 | 0.027 | 0.023 | 0.239 |
| 566 | 0.569 | -0.094 | 0.050 | 0.063 |
| 604 | 0.682 | 0.019 | 0.037 | 0.596 |
| 616 | 0.696 | 0.034 | 0.027 | 0.206 |
| 630 | 0.673 | 0.011 | 0.013 | 0.409 |
| 688 | 0.682 | 0.019 | 0.018 | 0.293 |
| 702 | 0.672 | 0.009 | 0.023 | 0.695 |
| 703 | 0.663 | 0.000 | 0.018 | 0.997 |
| 716 | 0.681 | 0.018 | 0.023 | 0.434 |
| 724 | 0.522 | -0.141 | 0.064 | 0.027 |
| 756 | 0.544 | -0.119 | 0.043 | 0.006 |
| 762 | 0.546 | -0.116 | 0.036 | 0.001 |
| 764 | 0.747 | 0.085 | 0.031 | 0.007 |
| 788 | 0.717 | 0.054 | 0.020 | 0.007 |
| 792 | 0.704 | 0.041 | 0.013 | 0.001 |
| 807 | 0.670 | 0.008 | 0.027 | 0.777 |
| 826 | 0.663 | 0.000 | 0.011 | 0.990 |
| 858 | 0.576 | -0.087 | 0.071 | 0.222 |
| 909 | 0.655 | -0.007 | 0.054 | 0.889 |

1. *Item: “Journalists provide fair coverage of elections”*

| **Countries flagged as invariant** | |
| --- | --- |
| Country Code | Country |
| 8 | Albania |
| 20 | Andorra |
| 31 | Azerbaijan |
| 32 | Argentina |
| 36 | Australia |
| 40 | Austria |
| 50 | Bangladesh |
| 51 | Armenia |
| 70 | Bosnia and Herzegovina |
| 100 | Bulgaria |
| 124 | Canada |
| 152 | Chile |
| 158 | Taiwan |
| 170 | Colombia |
| 191 | Croatia |
| 196 | Cyprus |
| 203 | Czechia |
| 208 | Denmark |
| 233 | Estonia |
| 246 | Finland |
| 250 | France |
| 276 | Germany |
| 300 | Greece |
| 320 | Guatemala |
| 344 | Hong Kong |
| 352 | Iceland |
| 356 | India |
| 364 | Iran |
| 368 | Iraq |
| 398 | Kazakhstan |
| 410 | South Korea |
| 417 | Kyrgyzstan |
| 422 | Lebanon |
| 428 | Latvia |
| 434 | Libya |
| 458 | Malaysia |
| 462 | Maldives |
| 484 | Mexico |
| 496 | Mongolia |
| 499 | Montenegro |
| 504 | Morocco |
| 554 | New Zealand |
| 566 | Nigeria |
| 578 | Norway |
| 586 | Pakistan |
| 604 | Peru |
| 608 | Philippines |
| 620 | Portugal |
| 688 | Serbia |
| 702 | Singapore |
| 703 | Slovakia |
| 705 | Slovenia |
| 716 | Zimbabwe |
| 724 | Spain |
| 752 | Sweden |
| 756 | Switzerland |
| 762 | Tajikistan |
| 764 | Thailand |
| 788 | Tunisia |
| 804 | Ukraine |
| 807 | North Macedonia |
| 818 | Egypt |
| 826 | United Kingdom (Great Britain) |
| 858 | Uruguay |
| 860 | Uzbekistan |
| 909 | United Kingdom (Northern Ireland) |

Weighted Average Factor Score Across Invariant Groups:

0.541

| **Unstandardized factor weights for the invariant countries, and their differences from the average** | | | | |
| --- | --- | --- | --- | --- |
| Country Code | Value | Difference | SE | p |
| 8 | 0.572 | 0.031 | 0.039 | 0.427 |
| 20 | 0.486 | -0.055 | 0.046 | 0.239 |
| 31 | 0.583 | 0.042 | 0.015 | 0.005 |
| 32 | 0.358 | -0.183 | 0.066 | 0.006 |
| 36 | 0.455 | -0.086 | 0.042 | 0.039 |
| 40 | 0.472 | -0.069 | 0.038 | 0.072 |
| 50 | 0.528 | -0.013 | 0.026 | 0.624 |
| 51 | 0.555 | 0.015 | 0.022 | 0.501 |
| 70 | 0.562 | 0.021 | 0.020 | 0.297 |
| 100 | 0.529 | -0.012 | 0.021 | 0.580 |
| 124 | 0.544 | 0.003 | 0.012 | 0.825 |
| 152 | 0.590 | 0.049 | 0.019 | 0.010 |
| 158 | 0.198 | -0.342 | 0.147 | 0.020 |
| 170 | 0.421 | -0.120 | 0.052 | 0.021 |
| 191 | 0.440 | -0.101 | 0.032 | 0.002 |
| 196 | 0.536 | -0.005 | 0.023 | 0.842 |
| 422 | 0.554 | 0.013 | 0.021 | 0.535 |
| 818 | 0.560 | 0.019 | 0.016 | 0.223 |
| 368 | 0.546 | 0.005 | 0.010 | 0.633 |
| 203 | 0.567 | 0.026 | 0.013 | 0.039 |
| 208 | 0.608 | 0.067 | 0.027 | 0.012 |
| 233 | 0.448 | -0.093 | 0.037 | 0.011 |
| 246 | 0.587 | 0.047 | 0.0500 | 0.349 |
| 250 | 0.558 | 0.018 | 0.014 | 0.218 |
| 276 | 0.512 | -0.029 | 0.025 | 0.243 |
| 300 | 0.547 | 0.006 | 0.013 | 0.636 |
| 320 | 0.501 | -0.039 | 0.036 | 0.273 |
| 344 | 0.572 | 0.031 | 0.017 | 0.061 |
| 352 | 0.610 | 0.069 | 0.039 | 0.080 |
| 356 | 0.540 | -0.001 | 0.026 | 0.966 |
| 364 | 0.547 | 0.006 | 0.030 | 0.844 |
| 398 | 0.554 | 0.013 | 0.018 | 0.475 |
| 410 | 0.600 | 0.060 | 0.025 | 0.016 |
| 417 | 0.566 | 0.025 | 0.021 | 0.223 |
| 428 | 0.507 | -0.034 | 0.022 | 0.121 |
| 434 | 0.562 | 0.021 | 0.027 | 0.437 |
| 458 | 0.547 | 0.006 | 0.018 | 0.730 |
| 462 | 0.478 | -0.063 | 0.031 | 0.041 |
| 484 | 0.494 | -0.047 | 0.054 | 0.390 |
| 496 | 0.690 | 0.149 | 0.049 | 0.002 |
| 499 | 0.611 | 0.071 | 0.029 | 0.014 |
| 504 | 0.594 | 0.053 | 0.023 | 0.022 |
| 554 | 0.540 | -0.001 | 0.009 | 0.902 |
| 566 | 0.560 | 0.019 | 0.019 | 0.312 |
| 578 | 0.576 | 0.035 | 0.038 | 0.352 |
| 586 | 0.594 | 0.053 | 0.019 | 0.005 |
| 604 | 0.597 | 0.056 | 0.041 | 0.171 |
| 608 | 0.617 | 0.076 | 0.037 | 0.041 |
| 620 | 0.592 | 0.051 | 0.017 | 0.002 |
| 688 | 0.562 | 0.021 | 0.017 | 0.218 |
| 702 | 0.550 | 0.009 | 0.022 | 0.676 |
| 703 | 0.531 | -0.010 | 0.018 | 0.575 |
| 705 | 0.524 | -0.017 | 0.026 | 0.507 |
| 716 | 0.580 | 0.039 | 0.022 | 0.070 |
| 724 | 0.537 | -0.003 | 0.007 | 0.612 |
| 752 | 0.615 | 0.074 | 0.048 | 0.127 |
| 756 | 0.589 | 0.049 | 0.021 | 0.023 |
| 762 | 0.603 | 0.063 | 0.022 | 0.004 |
| 764 | 0.534 | -0.007 | 0.028 | 0.813 |
| 788 | 0.562 | 0.021 | 0.018 | 0.248 |
| 804 | 0.471 | -0.070 | 0.022 | 0.001 |
| 807 | 0.549 | 0.008 | 0.024 | 0.744 |
| 826 | 0.476 | -0.065 | 0.022 | 0.003 |
| 858 | 0.542 | 0.001 | 0.016 | 0.947 |
| 860 | 0.654 | 0.114 | 0.041 | 0.005 |
| 909 | 0.558 | 0.017 | 0.038 | 0.656 |

1. *Item “Election officials are fair”*

| **Countries flagged as invariant** | |
| --- | --- |
| Country Code | Country |
| 8 | Albania |
| 20 | Andorra |
| 32 | Argentina |
| 36 | Australia |
| 40 | Austria |
| 50 | Bangladesh |
| 51 | Armenia |
| 68 | Bolivia |
| 70 | Bosnia and Herzegovina |
| 76 | Brazil |
| 152 | Chile |
| 158 | Taiwan |
| 170 | Colombia |
| 191 | Croatia |
| 368 | Iraq |
| 208 | Denmark |
| 218 | Ecuador |
| 246 | Finland |
| 250 | France |
| 268 | Georgia |
| 276 | Germany |
| 300 | Greece |
| 320 | Guatemala |
| 344 | Hong Kong |
| 348 | Hungary |
| 352 | Iceland |
| 360 | Indonesia |
| 392 | Japan |
| 398 | Kazakhstan |
| 404 | Kenya |
| 410 | South Korea |
| 417 | Kyrgyzstan |
| 434 | Libya |
| 440 | Lithuania |
| 484 | Mexico |
| 554 | New Zealand |
| 558 | Nicaragua |
| 566 | Nigeria |
| 578 | Norway |
| 586 | Pakistan |
| 608 | Philippines |
| 616 | Poland |
| 620 | Portugal |
| 630 | Puerto Rico |
| 642 | Romania |
| 702 | Singapore |
| 703 | Slovakia |
| 724 | Spain |
| 752 | Sweden |
| 756 | Switzerland |
| 762 | Tajikistan |
| 792 | Turkey |
| 807 | North Macedonia |
| 826 | United Kingdom (Great Britain) |
| 840 | United States |
| 858 | Uruguay |
| 909 | United Kingdom (Northern Ireland) |

Weighted Average Factor Score Across Invariant Groups:

1.052

| **Unstandardized factor weights for the invariant countries, and their differences from the average** | | | | |
| --- | --- | --- | --- | --- |
| Country Code | Value | Difference | SE | p |
| 8 | 0.913 | -0.139 | 0.118 | 0.238 |
| 20 | 1.828 | 0.776 | 0.418 | 0.064 |
| 32 | 1.921 | 0.869 | 0.836 | 0.298 |
| 36 | 1.083 | 0.031 | 0.105 | 0.765 |
| 40 | 0.908 | -0.144 | 0.070 | 0.040 |
| 50 | 0.991 | -0.061 | 0.074 | 0.412 |
| 51 | 0.857 | -0.195 | 0.063 | 0.002 |
| 68 | 0.759 | -0.293 | 0.155 | 0.059 |
| 70 | 0.874 | -0.178 | 0.059 | 0.002 |
| 76 | 1.486 | 0.434 | 0.523 | 0.406 |
| 152 | 0.944 | -0.108 | 0.067 | 0.108 |
| 158 | 1.116 | 0.064 | 0.207 | 0.757 |
| 170 | 1.409 | 0.357 | 0.280 | 0.202 |
| 191 | 0.978 | -0.074 | 0.072 | 0.307 |
| 368 | 1.378 | 0.326 | 0.268 | 0.224 |
| 208 | 0.881 | -0.170 | 0.065 | 0.008 |
| 218 | 3.172 | 2.120 | 2.329 | 0.363 |
| 246 | 0.953 | -0.099 | 0.166 | 0.549 |
| 250 | 1.049 | -0.003 | 0.085 | 0.976 |
| 268 | 1.081 | 0.029 | 0.11 | 0.795 |
| 276 | 0.893 | -0.158 | 0.058 | 0.007 |
| 300 | 1.135 | 0.083 | 0.107 | 0.441 |
| 320 | 1.082 | 0.030 | 0.103 | 0.77 |
| 344 | 0.920 | -0.132 | 0.058 | 0.023 |
| 348 | 0.840 | -0.212 | 0.109 | 0.052 |
| 352 | 0.799 | -0.253 | 0.115 | 0.028 |
| 360 | 0.860 | -0.192 | 0.081 | 0.018 |
| 392 | 0.999 | -0.053 | 0.072 | 0.464 |
| 398 | 1.023 | -0.029 | 0.075 | 0.695 |
| 404 | 0.768 | -0.284 | 0.114 | 0.013 |
| 410 | 0.889 | -0.163 | 0.068 | 0.016 |
| 417 | 1.034 | -0.018 | 0.116 | 0.875 |
| 434 | 0.903 | -0.149 | 0.077 | 0.054 |
| 440 | 0.963 | -0.089 | 0.036 | 0.014 |
| 484 | 1.127 | 0.075 | 0.188 | 0.689 |
| 554 | 1.572 | 0.520 | 0.387 | 0.179 |
| 558 | 1.072 | 0.020 | 0.133 | 0.878 |
| 566 | 1.061 | 0.009 | 0.113 | 0.939 |
| 578 | 0.932 | -0.120 | 0.130 | 0.359 |
| 586 | 0.914 | -0.138 | 0.061 | 0.024 |
| 608 | 0.879 | -0.173 | 0.071 | 0.015 |
| 616 | 1.032 | -0.020 | 0.138 | 0.886 |
| 620 | 0.945 | -0.107 | 0.061 | 0.078 |
| 630 | 1.392 | 0.341 | 0.360 | 0.344 |
| 642 | 0.891 | -0.161 | 0.050 | 0.001 |
| 702 | 0.939 | -0.112 | 0.066 | 0.086 |
| 703 | 1.029 | -0.023 | 0.061 | 0.704 |
| 724 | 1.727 | 0.675 | 0.449 | 0.133 |
| 752 | 0.824 | -0.228 | 0.104 | 0.028 |
| 756 | 0.907 | -0.144 | 0.067 | 0.032 |
| 762 | 0.899 | -0.153 | 0.060 | 0.011 |
| 792 | 0.965 | -0.087 | 0.057 | 0.128 |
| 807 | 0.970 | -0.082 | 0.078 | 0.292 |
| 826 | 1.124 | 0.072 | 0.067 | 0.284 |
| 840 | 0.914 | -0.138 | 0.054 | 0.011 |
| 858 | 1.465 | 0.413 | 0.326 | 0.206 |
| 909 | 1.047 | -0.005 | 0.143 | 0.972 |

**Countries flagged as invariant in terms of metric invariance at all three items:**

| **Country Code** | **Country** |
| --- | --- |
| 8 | Albania |
| 20 | Andorra |
| 32 | Argentina |
| 36 | Australia |
| 40 | Austria |
| 50 | Bangladesh |
| 51 | Armenia |
| 70 | Bosnia and Herzegovina |
| 170 | Colombia |
| 191 | Croatia |
| 208 | Denmark |
| 246 | Finland |
| 250 | France |
| 276 | Germany |
| 300 | Greece |
| 320 | Guatemala |
| 344 | Hong Kong |
| 368 | Iraq |
| 398 | Kazakhstan |
| 410 | South Korea |
| 417 | Kyrgyzstan |
| 434 | Libya |
| 484 | Mexico |
| 554 | New Zealand |
| 566 | Nigeria |
| 702 | Singapore |
| 703 | Slovakia |
| 724 | Spain |
| 756 | Switzerland |
| 762 | Tajikistan |
| 807 | North Macedonia |
| 826 | United Kingdom (Great Britain) |
| 858 | Uruguay |
| 909 | United Kingdom (Northern Ireland) |

1. ISO‑3166 numeric codes used in the World Values Survey databases [↑](#footnote-ref-1)
